# A Hopeful Sea-Monster: A Very Large Homologous Recombination Event Impacting the Core Genome of the Marine Pathogen *Vibrio anguillarum*

**DOI:** 10.3389/fmicb.2020.01430

**Published:** 2020-06-29

**Authors:** Nicola M. Coyle, Kerry L. Bartie, Sion C. Bayliss, Michaël Bekaert, Alexandra Adams, Stuart McMillan, David W. Verner-Jeffreys, Andrew P. Desbois, Edward J. Feil

**Affiliations:** ^1^The Milner Centre for Evolution, Department of Biology and Biochemistry, University of Bath, Bath, United Kingdom; ^2^Institute of Aquaculture, University of Stirling, Stirling, United Kingdom; ^3^Cefas Weymouth Laboratory, Weymouth, United Kingdom

**Keywords:** *Vibrio anguillarum*, whole genome sequencing, population structure, recombination, adaptation

## Abstract

*Vibrio anguillarum* is the causative agent of vibriosis in many species important to aquaculture. We generated whole genome sequence (WGS) data on a diverse collection of 64 *V. anguillarum* strains, which we supplemented with 41 publicly available genomes to produce a combined dataset of 105 strains. These WGS data resolved six major lineages (L1-L6), and the additional use of multilocus sequence analysis (MLSA) clarified the association of L1 with serotype O1 and *Salmonidae* hosts (salmon/trout), and L2 with serotypes O2a/O2b/O2c and *Gadidae* hosts (cod). Our analysis also revealed a large-scale homologous replacement of 526-kb of core genome in an L2 strain from a con-specific donor. Although the strains affected by this recombination event are exclusively associated with *Gadidae*, we find no clear genetic evidence that it has played a causal role in host specialism. Whilst it is established that *Vibrio* species freely recombine, to our knowledge this is the first report of a contiguous recombinational replacement of this magnitude in any *Vibrio* genome. We also note a smaller accessory region of high single nucleotide polymorphism (SNP) density and gene content variation that contains lipopolysaccharide biosynthesis genes which may play a role in determining serotype.

## Introduction

In common with many marine *Vibrio* species, *Vibrio anguillarum* is a commercially important pathogen of fish and shellfish, and is the causative agent of vibriosis in over 50 fish species world-wide (Frans et al., 2011). Infection is associated with the presence of several well-characterised virulence factors, including haemolysins, proteases and iron-uptake systems (Frans et al., 2011). The species is divided into at least 20 serotypes (Pedersen et al., 1999), but vibriosis in fish is predominantly caused by serotypes O1 and O2, and to a lesser extent serotype O3. The 20 remaining *V. anguillarum* serotypes are most probably environmental isolates from sediment, plankton or seawater, and these are considered mainly to be non-pathogenic (Austin et al., 1995, 1997). Vaccines are available for the main disease-causing serotypes O1, O2, and O3, although these do not protect against all O2 isolates, nor against the 20 other serotypes (Mikkelsen et al., 2007).

Molecular typing methods have been used to determine the population structure of this species and to identify major disease-causing clones. Steinum et al. (2016) developed a Multilocus Sequence Analysis (MLSA) scheme based on eight loci, and validated this on a diverse sample of 116 isolates of *V. anguillarum* and the closely related species *Vibrio ordalii*. These data defined major clones within the *V. anguillarum* population that were broadly consistent with serotype. Serotype O2b isolates were notable for being highly homogenous and were mostly isolated from *Gadidae* (cod). *V. anguillarum* samples have also been characterised using whole genome sequencing (WGS), and these studies have generated evidence concerning virulence, population structure and genome diversity (Busschaert et al., 2015; Castillo et al., 2017; Holm et al., 2018).

Here, we further explore the population structure and diversity of this species by full genome sequencing 64 *V. anguillarum* isolates from diverse sources. This dataset approximately doubles the number of genomes available for this species and was used in combination with existing WGS and MLSA data. In addition to providing a robust and more representative phylogeny of the species, and the delineation of major lineages, these data also revealed a novel large-scale recombination event which has resulted in the homologous replacement of 526-kb of the core genome of chromosome 1.

To our knowledge, this is the first report of a large-scale recombination event in *Vibrio* genomes, although ostensibly similar events have been reported in other species, notably *Klebsiella pneumoniae, Streptococcus agalactiae*, and *Staphylococcus aureus* (Brochet et al., 2008; Holden et al., 2010; Chen et al., 2014). Whilst the adaptive relevance of such events remains mostly unclear, the resulting hybrid strains have been likened to “hopeful monsters” (Croucher and Klugman, 2014), a reference to Richard Goldschmidt’s non-Darwinian argument that evolution can proceed in “jumps,” brought about by sudden large-scale genomic change (Goldschmidt, 1933). Although in most cases rapid and dramatic changes to the genome are likely to be deleterious (hence ‘monsters’), occasionally such events may be highly adaptive and provide the means to exploit a new niche (hence, ‘hopeful’). We also describe a smaller region of accessory gene content variation and high single nucleotide polymorphism (SNP) density, which shows features consistent with a genomic island and likely to be relevant for defining the serotype of the strains.

## Materials and Methods

### DNA Extraction and Sequencing

Sixty-four isolates were selected from the collection held at the Institute of Aquaculture, University of Stirling (Austin et al., 1995, 1997). The strains cover a wide range of serotypes, geographic regions, host species, and sampling dates. Each isolate was cultured from a single colony in 1.5% (w/v) NaCl-supplemented tryptone soya broth (TSB; Oxoid, Basingstoke, UK) to late exponential phase (approximately 14 h; 22°C; 150 rpm). Cells in 1 mL of each culture were collected by centrifugation and the DNA extracted by a salt precipitation method (Bartie et al., 2020). Libraries were generated using the Nextera XT kit (Illumina) and paired-end sequencing was performed on the Illumina MiSeq platform using a V3 kit with read length of 300-bp. Short reads have been deposited in the ENA archive under project accession number *PRJEB37012*.

Forty-one assemblies available on the NCBI were downloaded and added to the collection. References and accessions for each publicly available assembly can be found in the complete isolate list given in Supplementary Table S1.

### Mapping, Assembly, Quality Control (QC)

Raw sequence reads were trimmed using trimmomatic-0.36 (Bolger et al., 2014) with the following parameters: (ILLUMINACLIP:PE_All.fasta:2:30:10 SLIDINGWINDOW:4:20 MINLEN:36 TOPHRED33). Trimmed reads were quality tested using FastQC v0.11.7 (Andrews, 2010). Assemblies were made using SPAdes v3.11.1 with parameters [-k 55,77,87,99,107,117,127 –careful –only assembler]. Coverage per contig was calculated using BWA and SAMtools v1.8 (Li and Durbin, 2009). Contigs with coverage less than five and length less than 500-bp were removed. Assembly annotations were retrieved using prokka 1.13 with parameters [–addgenes –centre XXX –mincontiglen 200 –cdsrnaolap] (Seemann, 2014). QUAST v4.6.3 was used to assess the quality of assemblies (Gurevich et al., 2013).

A core-genome SNP alignment was created by mapping trimmed reads and publicly available assemblies to reference genome ATCC 68554 (775) (Naka et al., 2011) using snippy-3.2-dev (Seemann, 2015) (settings: –mincov 10 –mapqual 60 –unmapped). Using an in-house Perl script, low coverage (less than 10X) bases that had been set to the corresponding reference base were replaced with an N. Alignments for chromosomes one and two were concatenated.

### Phylogenetic Analysis

A phylogenetic best-scoring maximum likelihood (ML) tree of this mapping alignment was constructed using RAxML 8.2.10 (Stamatakis, 2014) [raxmlHPC-PTHREADS with parameters -f a -m GTRGAMMA -p 12345 -x 12345 -# 100]. PhyML version 20160207 was used with default parameters to estimate the transition to transversion ratio (kappa) for the population alignment (Guindon and Gascuel, 2003). Using this kappa value and the best-scoring ML tree as a starting tree, we tested for recombination using ClonalFrameML (Didelot and Wilson, 2015). First, a standard model analysis was undertaken with parameters [-kappa 6.415224 -emsim 100] to estimate the initial values needed. Subsequently, a per-branch model analysis was run using parameters [-kappa 6.415224 -embrace true -embranch_dispersion 0.1 -initial_values “0.769622 0.00269074 0.00269074”]. To mask regions of recombination in the alignment, clonal_frame_masker.sh was used (Kwong, 2018). RAxML was used to infer a new ML tree based on this masked alignment and using the same parameters as above. Trees were visualised and midpoint rooted in Figtree (Rambaut, 2016).

### Lineage Assignment

To assign isolates to lineages we used PopPUNK 1.1.2, which was run using k-mers (15, 19, 23, 27) (Lees et al., 2019).

### Pangenome Analysis

We used PIRATE (Bayliss et al., 2019) to build a comprehensive pangenome of the population and identify orthologous genes. Analysis of pangenome outputs was conducted using R version 3.2.3 (R Core Team, 2015; Wickham et al., 2019).

### *In silico* Multilocus Sequence Analysis (MLSA)

To compare the WGS dataset compiled here against a previously assessed MLSA dataset, we built a phylogenetic tree adding these WGS isolates to the existing MLSA sequence alignment. MLSA allele sequences were extracted from the 105 WGS assemblies using orthologs identified by PIRATE corresponding to the MLSA loci used by Steinum et al. (2016). One sequence was selected for each isolate that occurs in the MLSA study that has subsequently been sequenced. MLSA loci sequences were aligned using MAFFT. Gene alignments were trimmed to the length of corresponding loci using seqkit after visualising in SeaView (Gouy et al., 2010; Shen et al., 2016). A concatenated alignment of all eight loci was used to construct a maximum likelihood tree using RAxML 8.2.10 [raxmlHPC -f a -# 100 -m GTRGAMMA] (Stamatakis, 2014). Individual gene trees were generated using FastTree version 2.1.10 (Price et al., 2010; Katoh and Standley, 2013). Gene alignments were trimmed to the length of corresponding loci using seqkit after visualising in SeaView (Gouy et al., 2010; Shen et al., 2016). A concatenated alignment of all eight loci was used to construct a maximum likelihood tree using RAxML 8.2.10 [raxmlHPC -f a -# 100 -m GTRGAMMA] (Stamatakis, 2014). Individual genes trees were generated using FastTree version 2.1.10 (Price et al., 2010).

Trees and metadata were visualised using Microreact (Argimón et al., 2016) and can be accessed at the following URLs: WGS pre-recombination removal^1^; WGS post-recombination removal^2^; MLSA^3^.

### Analysis of Recombination

All isolates were mapped and variants called against the complete genome of VIB43 (Holm et al., 2018) using snippy (Seemann, 2015). A sliding window of single nucleotide polymorphism (SNP) density was conducted using an in-house python script with Biopython (Cock et al., 2009). The number of SNPs, per 1000-bp window, was calculated for pairs of isolates and visualised in R version 3.2.3 (R Core Team, 2015; Wickham et al., 2019). To visualise SNPs against the reference isolate VIB43, we used Artemis (Carver et al., 2012). Tabix was used to extract sections of VCF files for more detailed characterisation of SNPs (Li, 2011). An in-house python script was used to count synonymous, non-synonymous and intergenic SNPs, as identified by SnpEff (Cingolani et al., 2012). For phylogenetic analysis of specific regions of the VIB43 genome, sequences were extracted from the whole alignment using SeqKit (Shen et al., 2016). RAxML was used to build trees of these sequences (Simonsen et al., 2008; Darling et al., 2010). To assess the synteny of this region across the species, we aligned five complete genomes from across the tree (VIB43, VIB12, M3, JLL237, and 775) using ProgressiveMauve (Darling et al., 2010). Artemis comparison tool was used to compare complete genomes of VIB43 and 775 (Carver et al., 2005). We visualised gene content variation within the localised region of gene content variation using gggenes (Wilkins, 2017). BLAST was used to compare sequences with the NCBI nucleotide and protein databases (Altschul et al., 1990).

All bioinformatics analysis was carried out on a virtual machine hosted by MRC-CLIMB (Connor et al., 2016).

## Results

### Whole Genome Sequencing and the Combined Database

We generated short-read paired-end data on the Illumina MiSeq platform for 64 isolates of *V. anguillarum* from archived collections held at the University of Stirling. A summary of the QC, mapping, SNP calling and assemblies (see section “Materials and Methods”) is given in Supplementary Table S2. The 64 *V. anguillarum* strains represent diverse serotypes, hosts and geographical sources, with the oldest isolate, NCMB 572, isolated from a rainbow trout in Japan in 1958, and the most recent, 5240-C2, isolated from a European Bass in Portugal in 2016. Whilst five of these strains had previously been sequenced, the remaining strains were chosen to supplement *V. anguillarum* genome data already in the public domain (Supplementary Table S1). For example, serotype O3 is known to pose a relatively high disease burden but, as only four isolates corresponding to this serotype have previously been sequenced (three from Chile and one from France (Castillo et al., 2017; Holm et al., 2018), we chose a further seven serotype O3 isolates to sequence from Denmark, Italy and Japan. The combined dataset of 105 fully sequenced strains of *V. anguillarum*, including 41 publicly available sequences, represent at least 17 host species, 14 serotypes, and were isolated from North and South America, Europe, Asia and Australia over a 60-year time span (between 1958 and 2018). These metadata for all 105 WGS strains are available via the Microreact project^1^.

We further expanded our analyses through *in silico* MLSA of the sequenced isolates in order to draw comparisons with the data of Steinum et al. (2016), who characterised 110 diverse *V. anguillarum* and six *V. ordalii* isolates on the basis of eight housekeeping gene sequences. After excluding strains that were not clearly *V. anguillarum*, and those MLSA strains for which WGS data was also available, we used MLSA data for 84 additional strains. Metadata for all 189 strains (105 WGS plus 84 MLSA) are available via the Microreact project^3^, and summarised in Supplementary Table S1 and Figure S1.

### Phylogenetic Analysis, Lineage Assignment and Host Associations

**FIGURE 1:**
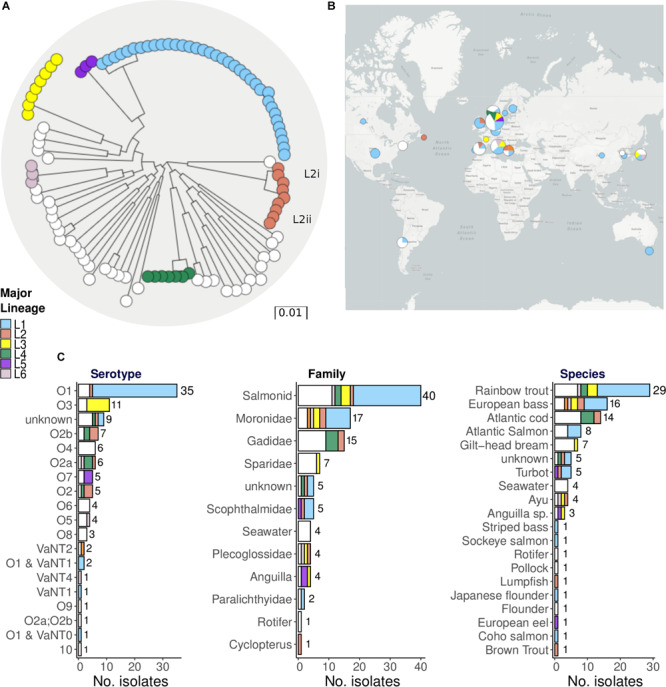
Phylogeny and metadata summary of 105 *Vibrio anguillarum* isolates highlighting major lineages. **(A)** Maximum-Likelihood phylogeny of 105 *Vibrio anguillarum* strains constructed using core SNPs mapped to the reference genome ATCC 68554 (775). The colours of the terminal nodes correspond to the six major lineages, identified by PopPUNK containing a minimum of three strains. Strains from the 31 minor lineages are all shown in white. Blocks of recombination have not been removed prior to reconstruction of this tree. **(B)** The proportion of isolates belonging to each major lineage according to geographical source. Pie charts are weighted by number of isolates. **(C)** Bar graphs indicate the number of isolates found with each serotype and host / environmental source. Hosts are grouped both by species and by family.

Figure 1A shows a maximum-likelihood phylogenetic tree of the 105 fully sequenced strains constructed using RaxML v8.2.10, based on core genome SNPs identified by mapping the short reads to reference strain ATCC 68554 (775), as described in the section “Materials and Methods.” This tree is free to explore in the Microreact project along with metadata and spatial data^1^. A bootstrapped version of this tree is given in Supplementary Figure S2. We used PopPunk (Lees et al., 2019) to identify 37 unique lineages, including six major lineages each with a minimum of three isolates (Supplementary Figure S3). PopPunk is a recently described K-mer based method for bacterial intra-species lineage assignment that incorporates variation in both the core and non-core genomes. In order to assess the impact of recombination, and the degree to which this has confounded the phylogenetic analysis and lineage assignment, we also analysed the data using ClonalFrameML (Didelot and Wilson, 2015), removed blocks of recombination, and then reconstructed the tree (see section “Materials and Methods”). Recombination events identified by ClonalFrameML are shown in Supplementary Figure S4, and the full output is given in Supplementary Table S5. Comparisons of the trees before and after the removal of recombination are given in Supplementary Figure S5, and the tree constructed after the removal of recombination can be explored via the Microreact project^2^.

Although the removal of recombination does not alter the delineation of the lineages, this procedure does alter branch lengths and changes the relationships between the lineages. We note that the branch leading to L1 has not been so clearly truncated by the removal of recombination, indicating that recombination may not have impacted as much on this lineage as the others.

Approximately one-third of the WGS isolates (36 of 105) correspond to a single major lineage, L1, and these isolates are predominantly serotype O1 strains (Figure 1C). Although most O1 isolates correspond to L1, there are exceptions such as the O1 isolate VIB43 which corresponds to L2 (Figure 1C), and four distantly related isolates (90-11-286, JLL237, S3 4/9, S2 2/9). These O1 isolates that do not correspond to L1 may reflect serotype switching, a phenomenon that is frequently associated with recombination. The next most common serotype is O3, which is associated with lineage L3. In contrast, lineages L2 and L4 are associated with multiple serotypes, indicating frequent serotype switching. No clear geographical or temporal patterns are discerned with respect to the distribution of these different lineages (Figure 1B and Supplementary Figure S6). There is some indication that L3 is mostly prevalent in Europe, but this group is represented by only seven European isolates (from Denmark, France and Italy) and only one non-European isolate (from Japan).

There are also hints in these data that different lineages might be associated with different hosts (Figure 1C). For example, L1 tends to be associated with *Salmonidae* and *Moronidae* (bass), whereas strains isolated from *Gadidae* (cod) are only found in major lineages L2 and L4, and some minor lineages. An examination of the subtree for L1 (with recombination removed) also pointed to the possibility of host association (Supplementary Figure S7). For example, L1 sub-lineages are evident that are associated with *Salmonidae* hosts, such as the cluster of related isolates associated with rainbow trout indicated by the curly red bracket in Supplementary Figure S7. However, potential host effects are difficult to disentangle from geographical structuring at this fine scale, as isolates in this cluster were all isolated from Denmark and Germany. Subtrees for the other lineages are also provided in Supplementary Figure S7 and can be explored with and without recombination removed via the Microreact URLs given above.

### Additional Evidence From MLSA Data

**FIGURE 2:**
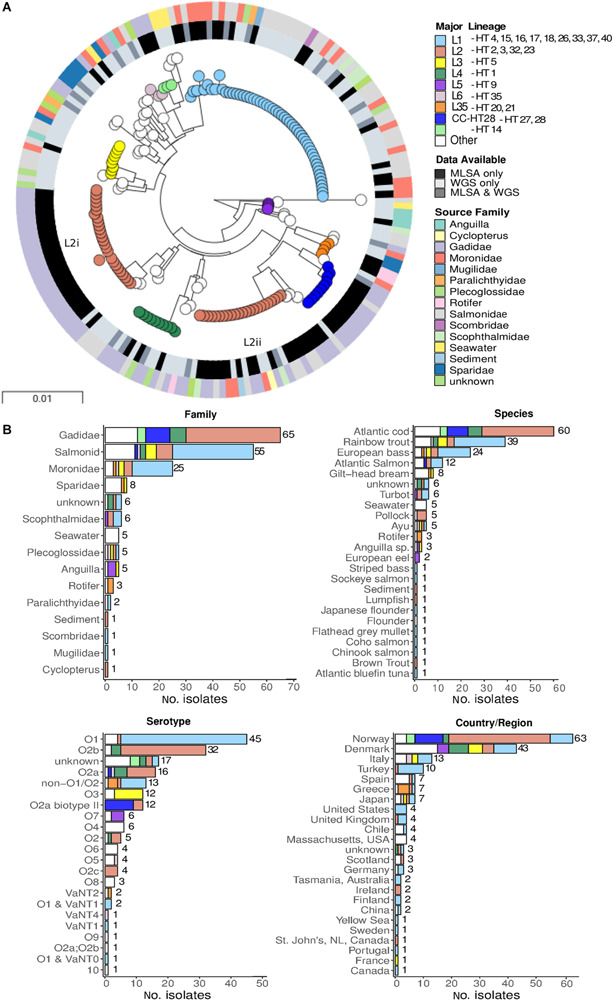
Phylogeny of 189 isolates based on eight MLSA loci. **(A)** Maximum likelihood phylogeny of 189 isolates based on the concatenated sequence of eight MLSA loci. Major lineages in this dataset were assigned by comparing PopPUNK assignments generated in this study and haplotypes (HT) assigned in Steinum et al. (2016). The inner ring represents the type of data are available for each isolate. As some isolates have been both genome sequenced and assessed using MLSA, these data are represented once in the tree (WGS & MLSA). **(B)** Metadata summary.

In order to place the major lineages defined using WGS data into a wider population context, we extracted MLSA loci sequences used by Steinum et al. (2016) from these genome data of the 105 WGS strains (see section “Materials and Methods”; total length 5236-bp), and produced a tree of the combined WGS+MLSA datasets for 189 isolates (Figure 2)^3^. With the notable exception of the L2 lineage (discussed below), these MLSA data resolved the same six major lineages (L1–L6) as WGS, but the increase in sample size means that additional lineages are also resolved. Supplementary Table S3 lists the WGS and MLSA major lineages for cross-referencing. The use of these MLSA data adds support to the view that the L1 lineage is characterised by isolates recovered from *Salmonidae* (*n* = 31, 57%) and *Moronidae* (*n* = 15, 27%), along with a small number of isolates from *Scophthalmus* (turbot) (*n* = 3, 5.5%). Even after the addition of these MLSA data, the L1 lineage contains no isolates from *Gadidae*.

A striking discrepancy between the lineages defined by WGS and MLSA data is that the latter subdivide L2 into two distinct and divergent lineages (L2i and L2ii). On close inspection this division is also evident, although much more subtle, when the whole genome is considered in the nine L2 isolates for which WGS data is available (Figure 1A and Supplementary Figure S7). These two lineages were noted as distinct major clones by Steinum et al. (2016) based on the MLSA data, with L2i corresponding to the serotype O2b clade (23 isolates, including NVI 6099, predominantly HT-2), and L2ii corresponding to the 17 isolates (including RV22) belonging to the serotype O2a/O2a biotype II/O2b/O2c clade and predominantly HT-4. These two groups show differences in host specialism; whereas L2i is 100% associated with *Gadidae* (26/26 after removing one “unknown” isolate), only 9/24 (37.5%) of the L2ii isolates are associated with this host species.

In order to determine which of these MLSA loci are responsible for the division of L2 into two distinct groups, we generated and compared individual MLSA gene trees (Supplementary Figure S8). This revealed that L2 is split into the same two distinct lineages in three of the gene trees; *ftsZ*, *rpoA*, and *pyrH*. Supplementary Table S4 gives the average pairwise nucleotide diversity (π) for each of the MLSA genes; it is clear from this table that *ftsZ* is by far the most diverged gene, and hence is contributing most strongly to the split between L2i and L2ii in the MLSA phylogeny.

### L2 Isolates Have Encountered a Large Homologous Replacement

A parsimonious explanation for the atypical phylogenetic signal in *ftsZ*, *rpoA*, and *pyrH* is that they have all been affected by the same large recombination event. These three genes are located at the following positions on the VIB43 reference genome: 2517629–2518360 (*pyrH*); 2664926–2666143 (*ftsZ*), and 2849390–2850382 (*rpoA*), spanning a total region of 332,753-bp and with none of the other MLSA genes being interspersed between them. This is consistent with the hypothesis that these three genes have been impacted by a single large recombination event in some of the L2 isolates, which accounts for the division of this lineage on the basis of these MLSA data. This possibility is also indicated by an examination of the ClonalFrameML output for the WGS strains (Supplementary Figure S4), where a large block of recombination is evident in two strains HI618 and 4299 corresponding to L2i.

**FIGURE 3:**
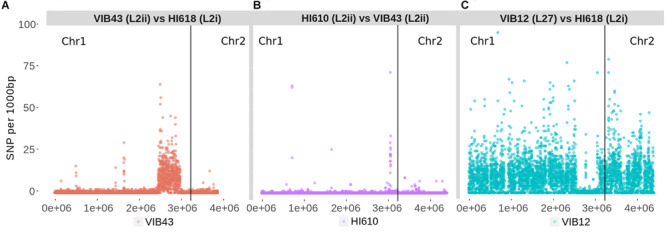
Number of SNPs per 1000-bp window for three pairwise comparisons showing the acquisition of a region of high SNP density in L2i strains from a lineage related to VIB12. SNPs were calculated based on variants called against chromosome 1 of VIB43. **(A)** VIB43 (L2ii) vs. HI618 (L2i) - a region of SNP density is present in the recombined region. **(B)** H610 (L2ii) vs. VIB43 (L2ii) – no region of SNP density. **(C)** VIB12 (L27) vs. HI618 (L2i) - a region of low SNP density is present in the recombined region.

**FIGURE 4:**
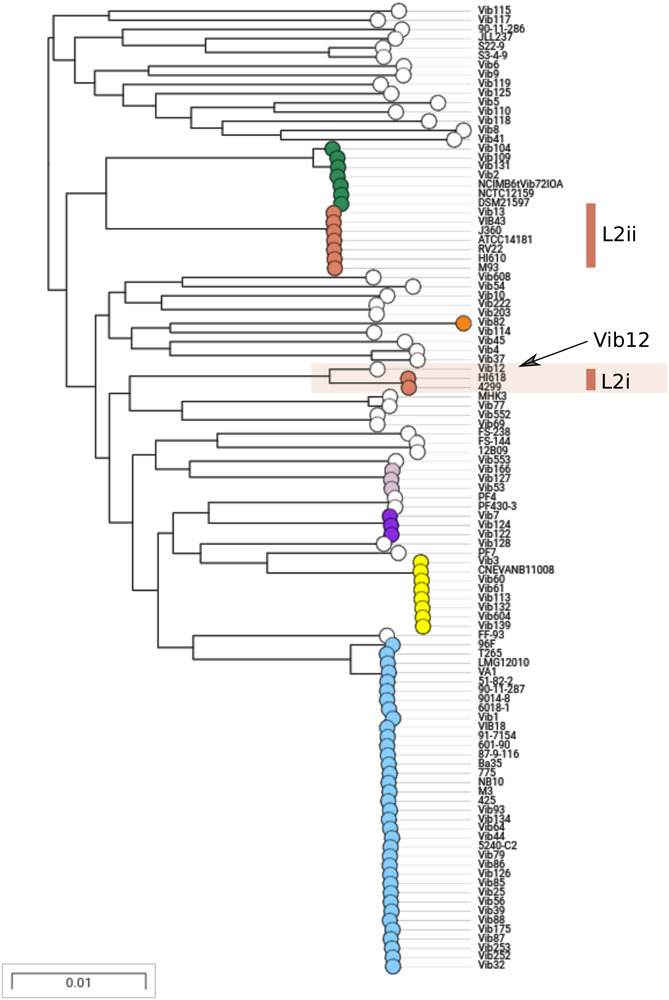
Maximum likelihood tree of all 105 isolates mapped to VIB43 based only on the 526-kb homologous recombination region. L2i and L2ii are found in distinct regions of the tree. L2i isolates are closely related to VIB12 in this region, implicating a relative of this strain as the donor.

We sought to further examine this hypothesis and to delineate the boundaries of the recombination block directly, by considering the distribution of SNPs between the L2i and the L2ii genomes. First, we plotted the number of SNPs within each 1-kb window between H610 vs. VIB43 (which both correspond to L2ii) and H618 vs. VIB43 (L2i vs. L2ii) (Figures 3A,B). This clearly confirmed the presence of a block of high-density SNPs of approximately 500-kb within chromosome 1 when HI618 was compared with VIB43 (L2i vs. L2ii), and this block of high SNP density is absent when the two L2ii strains are compared. In order to investigate the origin of the imported region in the L2 isolates, we constructed a tree of all the *V. anguillarum* isolates based only on the recombined sequence (Figure 4). As expected, this analysis completely separated the two sub-lineages L2i and L2ii. However, the two L2i strains cluster with isolate VIB12 (minor lineage L27), which reveals they have similar sequence within the recombined region. This implicates VIB12, or a close relative of this isolate, as the donor of the recombined region in L2i isolates. To confirm this, we plotted SNP density in 1-kb windows between isolates VIB12 and H618, which confirmed a high SNP density throughout most of chromosome 1, but much lower SNP density within the recombined region (Figure 3C). This analysis therefore confirms that L2i has been the recipient of a large replacement region donated from a close relative of VIB12.

**FIGURE 5:**
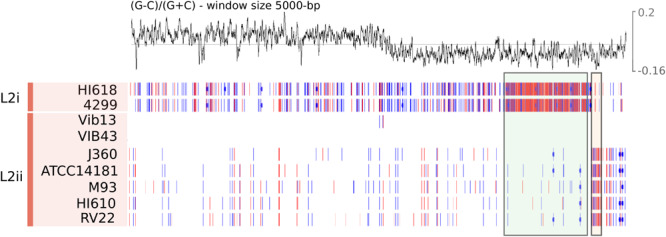
SNPs for each L2 isolate along chromosome 1 of VIB43 visualised using Artemis (Carver et al., 2012). Each line illustrates the synonymous (red) and nonsynonymous (blue) SNPs and complex multi-nucleotide variants (pink) for each isolate in L2. GC skew of VIB43 is shown above. A green box has been used to indicate the location of a large region of high SNP density between L2i isolates and VIB43. Similarly, a beige box highlights a smaller region of high SNP density between most L2ii isolates and VIB43.

In order to further delineate the recombined region, and to investigate the pattern of SNPs in chromosome 1 among all nine L2 isolates (two isolates for L2i and seven isolates for L2ii), we then mapped the short reads of the nine L2 genomes against VIB43, which is a fully closed genome corresponding to L2ii (Figure 5). This revealed the recombined region to be 525,878-bp long, with the average SNP density between L2i and L2ii genomes within this block of 0.85%, compared with 0.02% for the rest of chromosome 1. As expected, this region encompasses the three MLSA loci *ftsZ*, *rpoA*, and *pyrH*, thus explaining the atypical phylogenetic signal in these genes (Supplementary Figure S9). In contrast, the two L2ii strains HI610 and VIB43 only differed by 11 SNPs within the 525,787-bp recombined region, which is a typical level of diversity across the chromosome for these strains. The size of this replacement is similar to those previously reported for other species, most notably *S. aureus* (Holden et al., 2010) and *K. pneumoniae* (Chen et al., 2014), where the donors were also con-specific strains. However, to our knowledge this is the first time this phenomenon has been reported for *Vibrio* genomes.

We checked the relatedness between all nine WGS L2 isolates after the removal of recombination. As our analyses indicate that this recombination event is responsible for the division of L2 isolates into L2i and L2ii, we expected that the removal of this block would result in these two groups being indistinguishable. However, as in evident from Supplementary Figure S7, this was not the case; in fact, the two L2i isolates HI618 and 4299 remain distinct from the L2ii isolates, although the level of divergence is far lower than within the recombination block. The non-recombined SNPs accounting for the divergence between these lineages are broadly evenly distributed across the rest of chromosome 1. Assuming the large recombination event only happened once in the common ancestor of L2i, this divergence must have accrued over the rest of the genome subsequent to this recombination event. This raises the possibility that this recombination event may have resulted in ecological or genomic barriers to further gene flow. The observation from these MLSA data that L2i is more host-specialised towards *Gadidae* than L2ii is consistent with a degree of ecological separation between these lineages, thus supporting this hypothesis.

### SNPs Within the Recombined Region Have Experienced Purifying Selection

In order to examine the adaptive consequence of the large recombination event, we considered the pattern of synonymous and non-synonymous mutations within this region, which can provide evidence as to the strength and direction of natural selection. Rocha et al. (2006) noted that when highly closely related bacterial genomes are compared, the proportion of SNPs that are non-synonymous is greater than when more distantly related genomes are compared. For example, the SNPs between two isolates of *S. aureus* that belong to the same clonal complex will typically correspond to a dN/dS ratio of around 0.5, whereas this ratio will drop to approximately 0.1 when strains corresponding to different clonal complexes are compared (Castillo-Ramírez et al., 2011). This effect is due to a lag in purifying selection in removing slightly deleterious non-synonymous SNPs from the population. This means that more recently emerged SNPs are more likely to be non-synonymous than older SNPs. Castillo-Ramírez et al. (2011) showed that this effect can also explain patterns of dN/dS within single pairs of *S. aureus* genomes, where one of the genomes has been impacted by a large recombination event. Because the imported region originated from a diverged *S. aureus* isolate, the SNPs that were acquired on this region are older than other SNPs on the genome, and so have already passed through a selective filter in the donor chromosome. Thus, the dN/dS ratio within the recombined region is much lower than the rest of the genome.

We applied the same logic to check for purifying selection on the large recombination event in *V. anguillarum* L2 (Table 1). Similar to the analysis by Castillo-Ramírez et al. (2011), we note that there is a far higher proportion of synonymous SNPs within the diverged recombined region than in the rest of the chromosome (which is much more conserved as it has only very recently diverged between the two lineages). The majority of the 463 SNPs within chromosome 1 between strains H618 (L2i) and VIB43 (L2ii) outside of the recombination block are non-synonymous (*n* = 222) rather than synonymous (*n* = 170), which reflects the fact that these SNPs are recently emerged and not all of the slightly deleterious non-synonymous SNPs have been selectively purged. In contrast, of the 4,478 SNPs observed between these strains within the 525,878-bp block of recombination, non-synonymous SNPs are a minority (*n* = 675) compared to synonymous SNPs (*n* = 3422). The difference in proportions of synonymous and non-synonymous SNPs between the recombination block and the rest of the genome is highly statistically significant by a Fisher’s Exact Test (*P* < 0.0001), demonstrating that the dominant selective force acting on the SNPs within the recombined region has been purifying selection.

**TABLE 1:** SNP counts - synonymous, nonsynonymous and intergenic.

**Region**	**# sites**	**Total SNPs**	**% Divergence**	**# Syn SNPs**	**# Non-syn SNPs**	**# Intergenic SNPs**	**N/S**	**I/S**
Chromsome 1*	2496077	463	0.0185	170	222	71	1.31	0.42
Chromosome 2	1152743	161	0.0139	48	78	35	1.63	0.73
Recombination Block	525878	4478	0.852	3422	675	381	0.2	0.11

*Excluding the recombination block. Numbers of SNPs are calculated between genomes HI618 (L2i) and VIB43 (L2ii). N: ^#^Non-synonymous SNPs S: ^#^Synonymous SNPs I: ^#^Intergenic SNPs.

Thorpe and colleagues recently noted that the strength of purifying selection on intergenic sites is higher (on average) than on synonymous sites (Thorpe et al., 2017). This explains why there is also a significantly higher proportion of intergenic SNPs, relative to synonymous SNPs, outside the large recombination events compared to inside (*P* < 0.0001) (Table 1). Finally, we also note that the overall strength of purifying selection (as gauged by the N/S and I/S ratios) is higher in chromosome 1 than in chromosome 2. This is consistent with previous analyses (Dillon et al., 2017) and indicates that, despite the size of the event, the large recombination import that has impacted on chromosome 1 is likely to be conservative in terms of gene function. In order to examine this further, we considered the genes that have been affected by this event.

### The Recombined Region Mostly Affects Syntenic Core Genes

As discussed, there is an intriguing difference in host specificity between the two subdivisions of L2; all (26/26) of the L2i isolates are associated with *Gadidae* host, whereas only 37.5% (9/24) of the L2ii isolates were recovered from *Gadidae*. This raises the possibility that the large homologous replacement has an impact on host specialism. To investigate this, and to explore the adaptive relevance of this event more broadly, we compared the genes within the recombined region with those in the rest of the chromosome. First, we categorised each gene as either core (present in at least 95% of the genomes), or accessory (present in fewer than 95% of the genomes). Surprisingly, the recombined region is highly significantly enriched for core genes. Of the 464 genes within this region, 452 are core (97.41%). In contrast, when considering 1,874 genes within the non-recombined region of chromosome 1, 1,607 (85.86%) are core. This difference is highly significant (*P* < 0.0001; chi-sq. = 47.022, df = 1). We also note a lower proportion of core genes on chromosome 2 (596/784; 76.02%), which is again consistent with weaker purifying or stabilising selection acting on this replicon. As well as being enriched for core genes, the recombined block lies within a long collinear block, as identified by Progressive Mauve, indicating a high level of conserved synteny in the region within the population (Supplementary Figure S10).

We then used Shiny-GO (Ge et al., 2019) to compare the functional categories (gene ontologies) of the genes within the recombined region with those elsewhere in the genome (Table 2). The gene category that is most enriched within the recombined region are the ribosomal proteins, with 30 of the total complement of 56 being located within this region. These genes are the most conserved, and most highly expressed and hence *a priori* might be considered to be the least likely to undergo recombination. Other categories enriched within this region, including metabolic pathways and amino-acid biosynthesis, are also associated with essential housekeeping functions, thus we find no clear footprints of adaptation in terms of genes affected. However, it remains possible that allelic changes in core genes, or changes in how these genes are regulated, might have significant adaptive consequences.

**TABLE 2:** Enrichment of functional categories in recombination region 1 using ShinyGO.

**Functional category**	**FDR adjusted *P*-value**	**Genes in 0.5 Mb replacement**	**Total # genes in reference genome**	**Genes**
Ribosome	4.71–47	30	56	*rpmE rpsJ rplC rplD rplW rplB rpsS rplV rpsC rplP rpmC rpsQ rplN rplX rplE rpsN rpsH rplF rplR rpsE rpmD rplO rpmJ rpsM rpsK rpsS rplQ rpsB rpsI rplM*
Metabolic pathways	1.23–45	57	551	*pfka tpiA frdS frdC ppC argE argC argB argH aroK aroB purA rpoA cysD cysN cysC fbP ubiX murA arcB pepA gltX upP purM gmhA accA lpxB lpxA fabZ lpxD dxR pyrH thiL ribH proA proB gpT argA dapD thrB carB carA dapB lpxC murC murG murD mraY hldE enO pyrG mazG pdxJ rpiA leuA leuB leuC*
Biosynthesis of secondary metabolites	9.45–20	26	262	*pfkA tpiA frD frdC argE argC argB argH aroK aroB fbP ubiX arcB gltX purM accA dxR uppS gpT argA dapB enO rpiA leuA leuB leuC*
Biosynthesis of amino acids	9.45–20	20	119	*pfkA tpiA argE argC argB argH aroK aroB arcB proA proB argA dapD thrB dapB enO rpiA leuA leuB leuC*
Microbial metabolism in diverse environments	4.82–11	15	163	*pfkA tpiA frdD frdC ppC cysD cysN cysC fbP accA dapD thrB dapB enO rpiA*
Arginine and proline metabolism	3.07–10	8	28	*argE argC argB argH arcB proA proB argA*
2-Oxocarboxylic acid metabolism	8.68–10	8	32	*argE argC argB argH argA leuA leuB leuC*
Peptidoglycan biosynthesis	2.29–08	6	18	*murA murC murG murD mraY murE*
Lipopolysaccharide biosynthesis	2.96–08	6	19	*gmhA lpxB lpxA lpxD lpxC hldE*
Purine metabolism	4.79–08	9	76	*purA rpoA cysD cysN cysC purM gpT deoB mazG*
Flagellar assembly	6.53–08	7	37	*flgH flgG flgF flgE flgD flgC flgB*
Carbon metabolism	2.62–07	9	94	*pfkA tpiA frdD frdC ppC fbP accA enO rpiA*
Pyrimidine metabolism	8.44–07	7	54	*rpoA upP pyrH carB carA pyrG mazG*
Pentose phosphate pathway	1.74–06	5	21	*pfkA fbP deoB deoC rpiA*
Pyruvate metabolism	0.00000338	6	43	*frdD frdC ppC gloB accA leuA*
Methane metabolism	0.000067	4	22	*pfkA ppC fbP enO*
Aminoacyl-tRNA biosynthesis	0.0001	4	25	*epmA trpS valS gltX*
Glycolysis / Gluconeogenesis	0.00016	4	28	*pfkA tpiA fbP enO*
Alanine, aspartate and glutamate metabolism	0.0002	4	30	*argH purA carB carA*
Valine, leucine and isoleucine biosynthesis	0.00047	3	16	*leuA leuB leuC*
Lysine biosynthesis	0.00047	3	16	*dapD dapB murE*
RNA degradation	0.00048	3	16	*groL rnR enO*
D-Glutamine and D-glutamate metabolism	0.00063	2	4	*murC murD*
Protein export	0.00063	3	18	*secB secY secA*
Fructose and mannose metabolism	0.00080	3	20	*pfkA tpiA fbP*
Sulfur metabolism	0.00080	3	20	*cysD cysN cysC*
Oxidative phosphorylation	0.00230	3	29	*frdD frdC ppA*
Selenocompound metabolism	0.00230	2	8	*cysD cysN*
Two-component system	0.00230	6	154	*cpxA cpxR frdD frdC rpoN glnD*
Terpenoid backbone biosynthesis	0.00430	2	11	*dxr uppS*

Although the vast majority of the genes within the recombined region are core, a small number (*n* = 12) are accessory, and we checked if the presence/absence of these genes might be relevant for host specialism. Of these 12 genes, six are missing in both L2i strains while present in all L2ii strains. One of the genes missing in the recombined block contains Sel1-like repeats (SLR) repeats and shares weak homology (27.7% amino acid identity) with *esiB* in *E. coli* which has been implicated in immune evasion (Pastorello et al., 2013). In VIB43, this SLR containing gene (CK207_10440) is flanked by multiple IS66 family insertion sequences, which raises the possibility that it may be frequently gained and lost in the population, and it is adjacent to three other genes missing in L2i isolates, including luxR, the regulatory protein associated with quorum sensing (Chen and Xie, 2011). These genes all lie within a 15 gene segment that is missing in L2i (Supplementary Figure S11). Multiple copies of both *esiB* and *luxR* are found in the genome of VIB43 although close homologues of this copy are only present in 15 strains in our dataset, including L6 strains, Vib54 and Vib608.

### Gene Content and SNP Variation in Regions Neighbouring the Large Recombination Block

Whilst demarcating the boundaries of the large recombination event described above, we noted another localised region of high SNP density (relative to the VIB43 reference) of 14,280 bp located 71-kb from the large block of recombination towards the origin of replication on chromosome 1 (Figure 5). This 14-kb region in VIB43 lies within a longer 28,403-bp element that contains 25 genes. These 25 genes are variably present or absent in other strains and lineages and have previously been identified as playing a role in immune evasion (Castillo et al., 2017).

**FIGURE 6:**
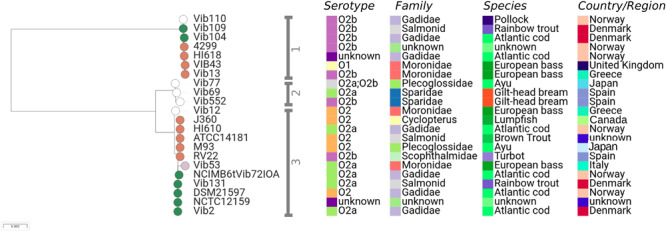
Tree constructed based on LPS biosynthesis genomic island (for the 22 isolates in which this region is present). Mapping to this segment (14,281-bp long) in VIB43 was used to build the tree. The tree distinguished three variants of this region, indicated by grey brackets. Variant 1 is associated with serotype O2b and is found in isolates from L2i, L2ii, L4 and isolate Vib10. Variant 2 is found in three distinct isolates Vib77, Vib69 and Vib552. Variant 3 is associated with multiple serotypes, and is found in isolates from lineages L2, L4, L6, and isolate VIB12.

Thus, in contrast to the large recombination event described above, the genes in this region are almost entirely accessory. We have identified 22 isolates with the complete, or near-complete, complement of these genes; these are: all isolates corresponding to L2 (*n* = 9) and to L4 (*n* = 7), a single isolate from L6 (Vib53), and five isolates from minor lineages (Vib110, VIB12, Vib77, Vib69 and Vib552). This region in the L1 reference genome 775 contains an entirely different suite of genes (Supplementary Figure S12). A nucleotide BLAST search of the 28-kb element revealed four regions of similarity to a lipopolysaccharide (LPS)-(O-antigen) biosynthesis-related sequence in *Vibrio* c*holerae* strain CO845 (Accession: GU576499.1. BLAST: 90.25% nucleotide id, 60% total query cover) (Aydanian et al., 2011). One of these four regions corresponds to the 14-kb region of high SNP density that is embedded within this element. Phylogenetic analysis of this 14-kb region resolves three variants (Figure 6). Variants 1 and 3 are most common and each correspond to a mixture of L2 and L4 strains, indicating lateral transfer between these lineages. Variant 1 is found mainly in isolates with sero-subtype O2b (5/7), whereas variants 2 and 3 are more varied in sero-subtype. The putative association of variant 1 and sero-subtype of O2b is consistent with the role of this region in the synthesis of surface antigens. A close inspection of the gene content within this 14-kb region reveals that in variant 1 strains (VIB43 in Supplementary Figure S13) *fmt* has been replaced with three genes, an ISVa15 family transposase, *pglE*, and a hypothetical protein with some similarity to sugar O-acetyltransferases. *pglE* is an important colonisation factor in other species (Schoenhofen et al., 2006), and encodes a UDP-N-acetylbacillosamine transaminase which is involved in protein glycosylation. As this gene is only present in isolates containing variant 1 of this gene cluster, it is possible that it plays a role in the synthesis of the serotype O2b reactive surface antigens. Castillo et al. (2017) also described a second smaller capsule-related gene cluster, adjacent to the one described above. This cluster is also likely to play a role in defining sero-subtypes, as described in Supplementary Information (Supplementary Figure S14).

## Discussion

Here, we describe a population genomics analysis using WGS data for 105 diverse isolates of the important aquaculture pathogen *V. anguillarum*. Sequence data for 64 of these strains were generated, although five of these had been previously sequenced. Publicly available WGS data for a further 41 strains were also used, to give a total of 105 genomes. Phylogenetic analysis revealed six major lineages, L1-L6, each represented by at least three isolates, and these were confirmed using PopPunk which simultaneously considers both core and non-core variation to delimit clusters. We strongly advocate the use of this approach for lineage assignment, particularly for species where multilocus sequence typing (MLST) schemes have not been developed. We used ClonalFrameML to detect and remove recombination events. The removal of recombination did not affect lineage assignments, but did alter the relationships between the lineages and truncate branch lengths, except in the branch leading to L1. Although the removal of recombination prior to phylogenetic analysis is a common approach, previous studies have urged caution as this can potentially decrease, rather than increase, the reliability of the tree (Saltykova et al., 2019).

There is a mixed picture regarding the strength of association between lineages and serotypes. Lineage L1 is almost exclusively associated with serotype O1, and L3 is strongly associated with O3; this suggests that serotype switching by recombination is rare in these lineages. In contrast, lineages L2 and L4 represent multiple sero-subtypes O2a/O2b/O2c, signifying more frequent switching events. These WGS data also hint at differences in host specialism between lineages L1 and L3, which tend to be associated with *Salmonidae* (trout/salmon) and *Moronidae* (bass); and lineages L2 and L4, which are associated with *Gadidae* (cod). The evidence for host specialisms between lineages is strengthened by the addition of MLSA data (Figure 2), which provides further support for associations between L1 and *Salmonidae* / *Moronidae*, and L2 with *Gadidae*.

Although these MLSA data are only based on eight gene loci, and thus has poor resolving power compared to the genome sequences, the inclusion of these data led to the serendipitous discovery of a very large homologous recombination event resulting in the “cut-and-paste” of over 526-kb of the core genome in chromosome 1, from a con-specific donor sequence (related to strain VIB12) to an L2 isolate. In our view, the discrepancy between in the MLSA and WGS data with regard to the L2 lineage does not reflect a weakness in MLSA *per se*, nor the choice of the MLSA genes for *V. anguillarum*, but simply results from a rare event that could not have been predicted and which just happened to have impacted on three of the MLSA genes in one specific lineage. It is tempting to intuit that such a large-scale genomic change must have significant consequences (either positive or negative) for cell fitness or adaptation (Mostowy et al., 2014); thus strains of *K. pneumoniae*, where such events are relatively common (Comandatore et al., 2019), have been likened to “hopeful monsters” (Croucher and Klugman, 2014). Regarding the event described in this present study, the ecological and genetic evidence regarding adaptive consequences are somewhat conflicting. The limited ecological evidence points to increased host specialisation within the L2i isolates that have inherited the recombination block; these are exclusively associated with *Gadidae* (26/26 strains) whereas only 9/24 (37.5%) of the L2ii isolates (which are closely related to L2i strains, but lacking the imported region) are associated with this host group. To test the true host range of L2i we would suggest extensive sequencing of isolates, particularly those with serotype O2b, from multiple hosts and geographic locations. If L2i is truly associated with *Gadidae* and O2b, we would expect only O2b isolates from *Gadidae* to lie within lineage L2i. Indirect genetic support for a host shift is provided by the observation that L2i and L2ii strains have begun to diverge elsewhere in the genome, which might be expected due to differing ecologies resulting in more restricted opportunities for gene transfer. On the other hand, the SNPs introduced by the large recombination event are mostly synonymous and, strikingly, the genes contained within this region are strongly enriched for conserved core functions, including ribosomal proteins and central metabolism. Comandatore et al. (2019) recently surveyed large recombinational replacements in *K. pneumoniae* and argued that large recombination events in regions close to the origin of replication, where essential genes reside in high density within the *Proteobacteria*, are likely to be deleterious. However, a simple explanation for the presence of this recombination block would be that conserved regions share the closest homology, which might mechanistically favour homologous recombination. Viewed from this perspective the large import is at best selectively tolerated, but most likely falls short of conferring a positive advantage. We do however stress that our failure to identify obvious candidate genes within the recombined region that can be readily implicated in host specialism does not mean that such changes have not occurred, as such ecological shifts can result from quite subtle changes in gene expression or regulation (Denef et al., 2010).

Although, to our knowledge, this is the first report of such a large-scale contiguous recombination event affecting *Vibrio* genomes, recombination is known to be frequent in *Vibrio* species and has been linked to ecological shifts (Efimov et al., 2013). Moreover, ostensibly similar large-scale recombination events have been reported for other bacterial species, including *Clostridium difficile* (He et al., 2013), *S. agalactiae* (Brochet et al., 2008) and *K. pneumoniae* (Holt et al., 2015). Robinson and Enright (2004) reported two such events affecting regions of the genome near the origin of replication in *S. aureus*, the largest of which (>550-kb) was characteristic of the important hospital-acquired methicillin resistant (MRSA) clone ST239. Despite an apparent fitness cost, evidenced by increased doubling time of this clone, ST239 was at one point the most common hospital-acquired MRSA strain globally, although it has largely been replaced by other strains over the last few years (Li et al., 2018). The extent to which the rise, or the subsequent fall, of this clone can be attributed to the large recombination event remains unclear.

Other large-scale recombination events have been described for *S. aureus* (Didelot and Wilson, 2015; Nimmo et al., 2015; Aanensen et al., 2016), but the most convincing example of a causal link between such an event and host specialisation was described by Spoor et al. (2015). In this case, recombination affected a 329-kb region spanning the origin of replication and resulting in the hybrid bovine-adapted *S. aureus* clone ST71. The large replacement in this clone (which was itself mosaic in origin) led to loss of human-adapted genes and the gain of bovine-adapted genes, probably originating from pre-existing bovine adapted lineages.

In addition to the large recombination event, we also examined diversity within two LPS and capsule synthesis gene clusters previously identified by Castillo et al. (2017), which are positioned approximately 70-kb from the large recombination block. LPS is known to play an important role in immune evasion and adherence in *V. anguillarum* (Lindell et al., 2012), and the expression of genes coding for LPS production, transport and assembly is linked to environmental stresses such as low temperature and low iron availability (Lages et al., 2019). Here, we also note associations between gene content and nucleotide variation in these elements with serotype definition. Most notably, one cluster containing 25 genes in VIB43 is found almost exclusively in serotype O2 strains, and has striking similarities to a LPS-biosynthesis related gene locus in *V. cholerae* (Aydanian et al., 2011). Intra-species transfer of LPS-biosynthesis genes has previously been linked to the formation of entirely new serotypes in *V. cholerae* (Yamasaki et al., 1999). In some cases, genes in this locus show greater similarity to *Vibrio* species other than *V. cholerae*, pointing to inter-species transfer of these gene cassettes. A final question remains as to whether the presence of these variable, and presumably mobile, gene cassettes is linked to the large recombinational replacement. The possibility of such a link is raised by the work of Brochet et al. (2008), who noted that *S. agalactiae* strains exchange large regions of DNA through *cis-* and *trans-* mobilisation by conjugative elements. However, further detailed comparative genomic and experimental work is required to test this hypothesis.

## Data Availability Statement

The datasets generated for this study can be found in the NCBI with Project Code PRJEB37012.

## Author Contributions

EF, DV-J, AA, and AD designed the study. KB, MB, and SM carried out sequencing and microbiology. NC carried out the bioinformatics analysis, with input from SB. EF and NC wrote the manuscript with input from all authors. All authors contributed to the article and approved the submitted version.

## Conflict of Interest

The authors declare that the research was conducted in the absence of any commercial or financial relationships that could be construed as a potential conflict of interest.

## Footnotes

1 https://microreact.org/project/X-2CDGKN1

2 https://microreact.org/project/F0V23AIZW

3 https://microreact.org/project/gjI4aftM1

## Supplementary Material

FIGURE S1 Summary of 189 strains used in this study divided based on where the data was sourced.

FIGURE S2 Pre-recombination removal ML tree of 105 isolates with bootstrap values.

FIGURE S3 37 PopPUNK lineages were identified in 105 *Vibrio anguillarum* isolates.

FIGURE S4 ClonalFrameML output for 105 *V. anguillarum* isolates based on SNP alignment against the complete genome ATCC 68554 (775).

FIGURE S5 Comparison of phylogeny pre and post recombination removal.

FIGURE S6 Timeline of WGS isolate sampling.

FIGURE S7 Subtrees of 6 major lineages (post-recombination removal) with corresponding metadata.

FIGURE S8 Individual trees for 8 MLSA loci constructed using FASTtree.

FIGURE S9 Illustration of positions of MLSA loci on *Vibrio anguillarum* VIB43 chromosome 1.

FIGURE S10 ProgressiveMauve alignment of five complete genomes reveal that the large homologous replacement occurs on a long Locally Collinear Block (LCB).

FIGURE S11 Genes within the large homologous recombination absent in L2i.

FIGURE S12 Artemis Comparison Tool comparison of L2ii strain VIB43 and L1 strain 775, reveals variation in LPS biosynthesis related region of chromosome one.

FIGURE S13 Comparison of gene content on and beside the SNP dense region of the LPS biosynthesis-related genomic island in five representative isolates.

FIGURE S14 Tree produced using LPS and capsule related genomic island for the 36 isolates that carry this accessory region.

TABLE S1 Metadata for all 189 strains.

TABLE S2 Quality Control figures for read QC, mapping and assembly of 105 WGS isolates.

TABLE S3 Summary of 10 major lineages identified using WGS and MLSA.

TABLE S4 Average pairwise diversity for MLSA loci for 189 isolates.

TABLE S5 Clonal Frame output.
