# Supplementary material for: A Hopeful Sea-Monster: A Very Large Homologous Recombination Event Impacting the Core Genome of the Marine Pathogen Vibrio anguillarum
Source: Front Microbiol. 2020 Jun 29;11:1430. doi: 10.3389/fmicb.2020.01430 (PMC7336808; doi:10.3389/fmicb.2020.01430)
Supplement: FIGURE S1 — Summary of 189 strains used in this study divided based on where the data was sourced. [file Data_Sheet_1.PDF]

Supplementary Material

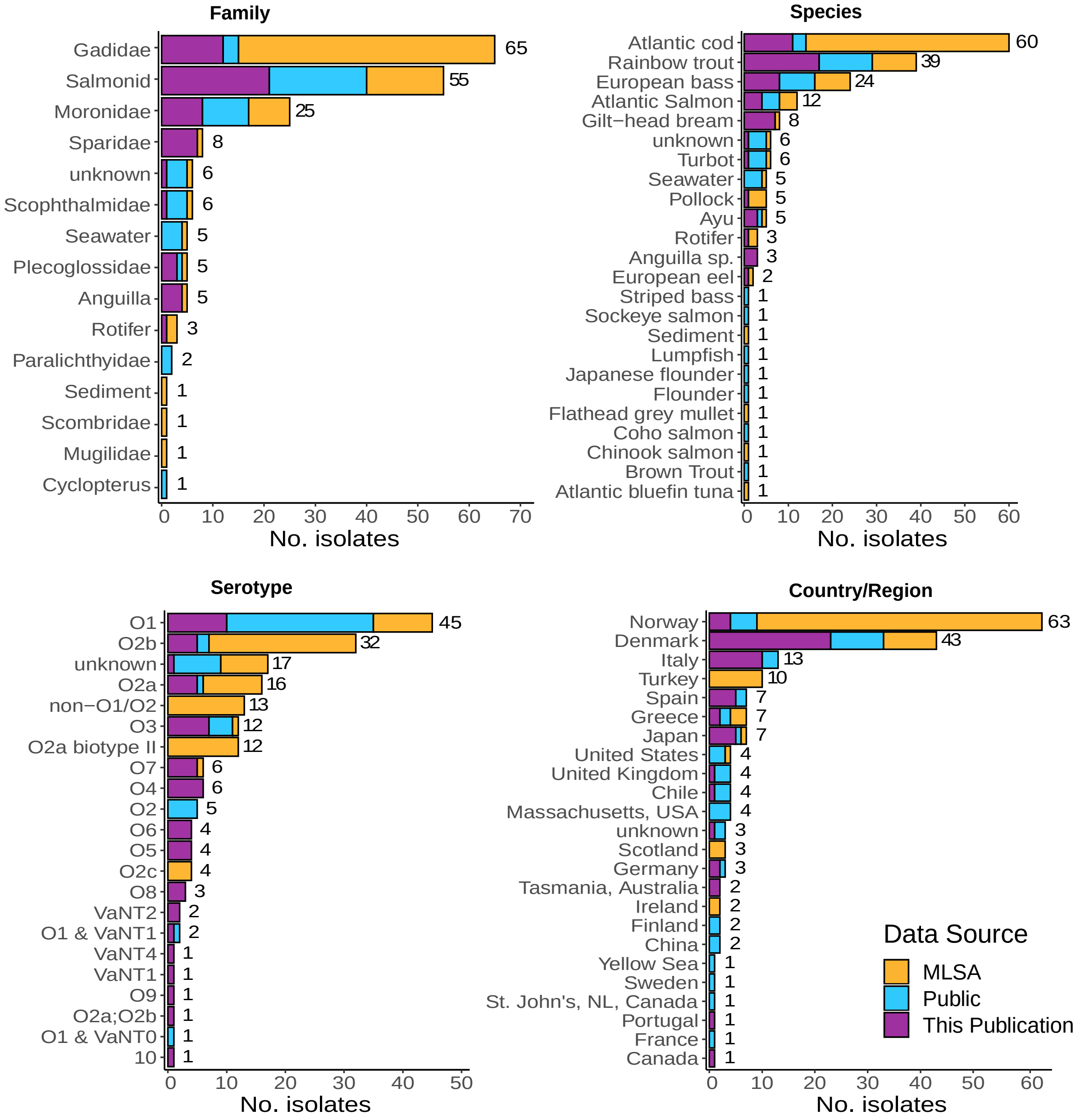

Figure S1: Summary of 189 strains used in this study divided based on where the data was sourced. Isolates sequenced in this publication were selected to cover a variety of serotype, geographic origin and source of isolation.

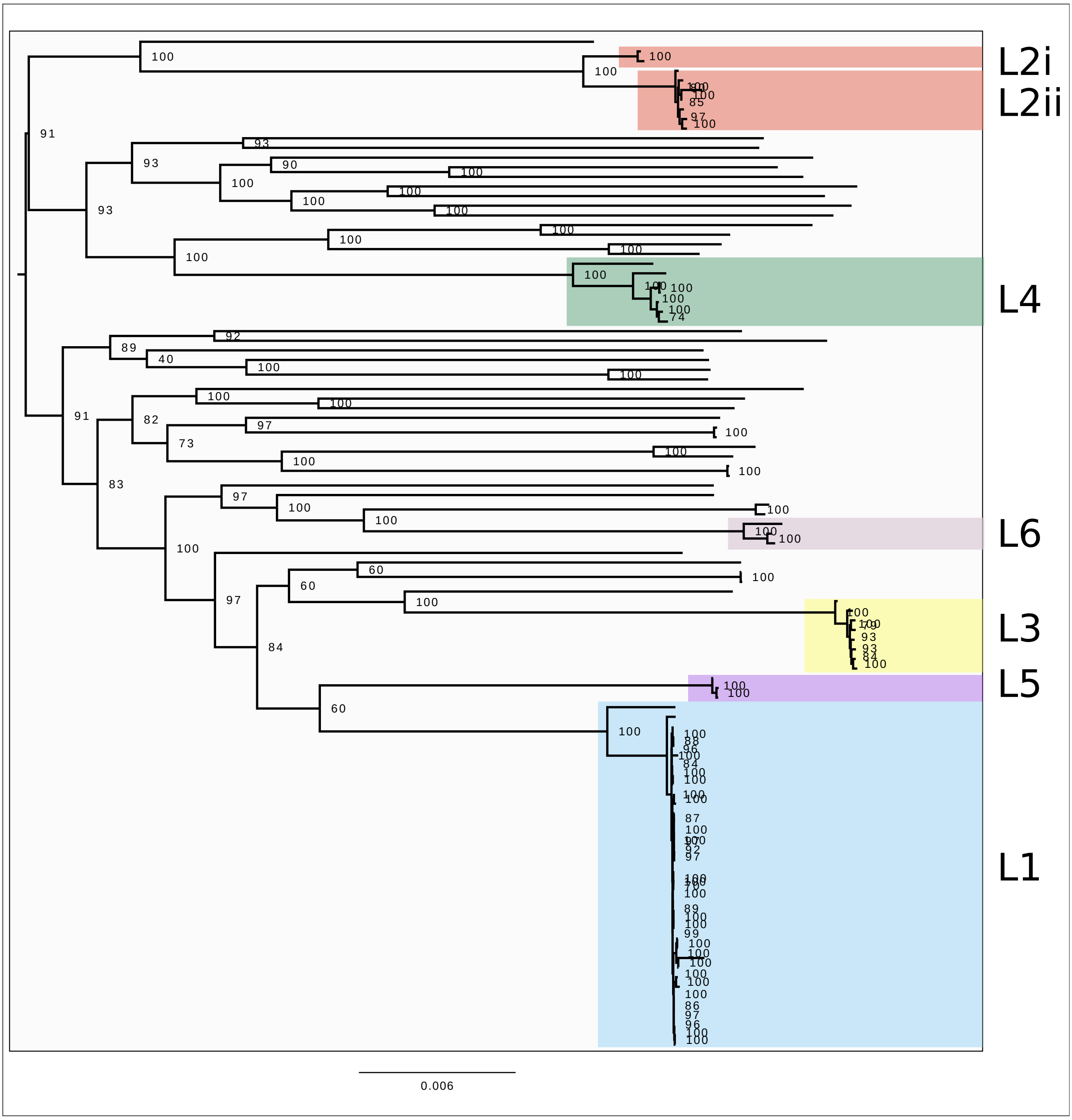

Figure S2: Tree of 105 isolates with bootstrap values. The six major lineages are coloured and labelled. All major linegaes are supported by 100% bootstrap values.

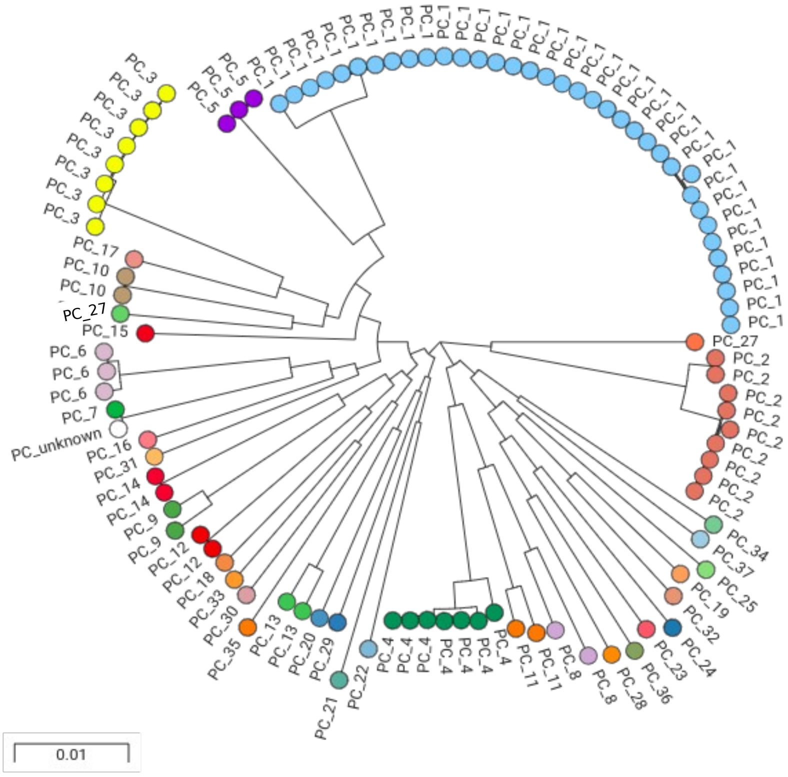

Figure S3: 37 PopPUNK lineages were identified in 105 *Vibrio anguillarum* isolates. Maximum-Likelihood phylogeny of 105 isolates with each leaf coloured and labelled by PopPUNK lineage.

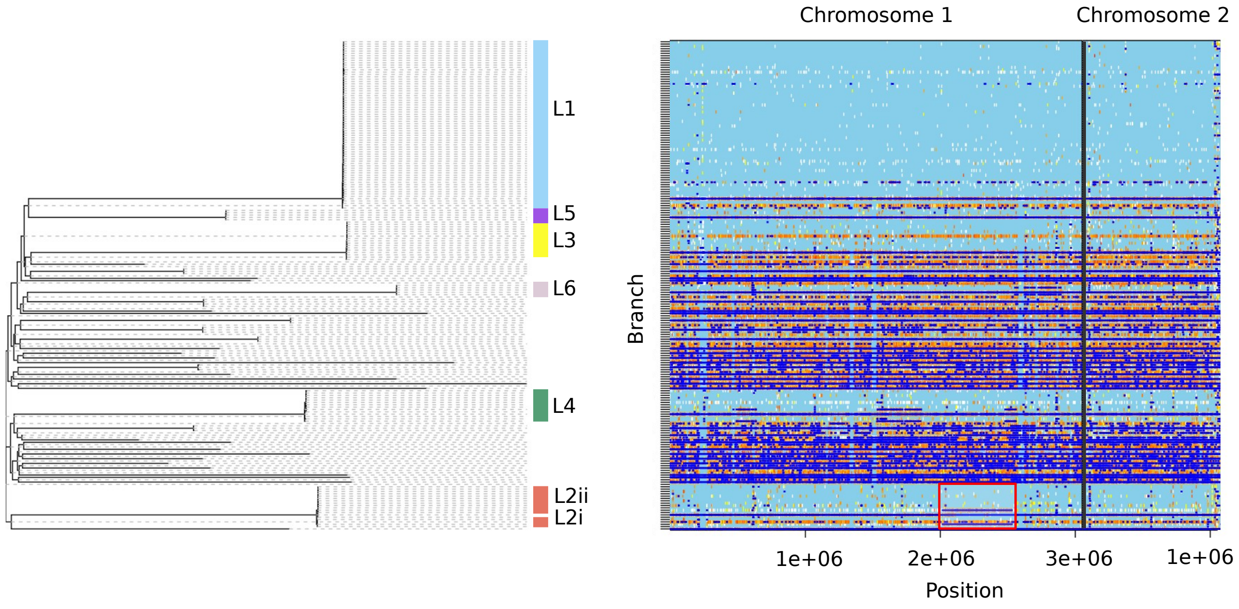

Figure S4: ClonalFrameML output for 105 *V. anguillarum* isolates based on SNP alignment against the complete genome ATCC 68554 (775). Major lineages on the tree are coloured. Each line represents a lineage or a node in the tree. Recombined sites are labelled in dark blue. Polymorphic sites are shown on a scale from white to red, depending on how closely congruent they are to the branching order on the tree - white for highly congruent sites, red for poorly congruent sites. A red box indicates the location of a large recombination region and the L2 isolates affected.

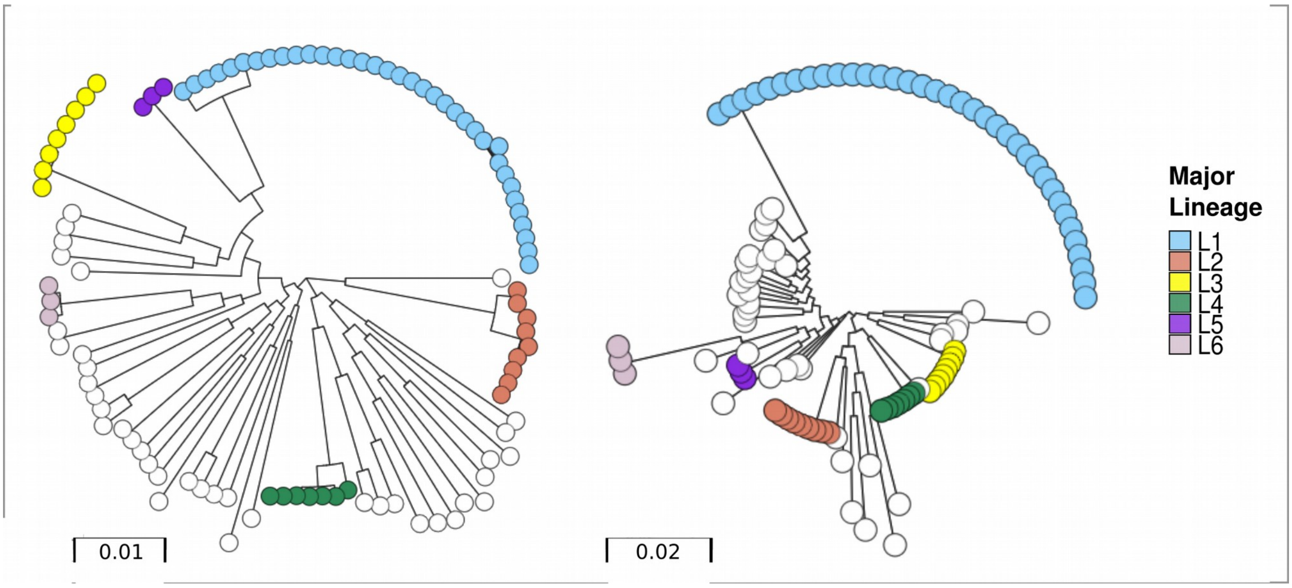

Figure S5: Comparison of phylogeny pre and post recombination removal. The trees are free to explore via the Microreact URLs: https://microreact.org/project/X-2CDGKN1 (Pre-recombination removal), and https://microreact.org/project/F0V23AIZW (Post recombination removal).

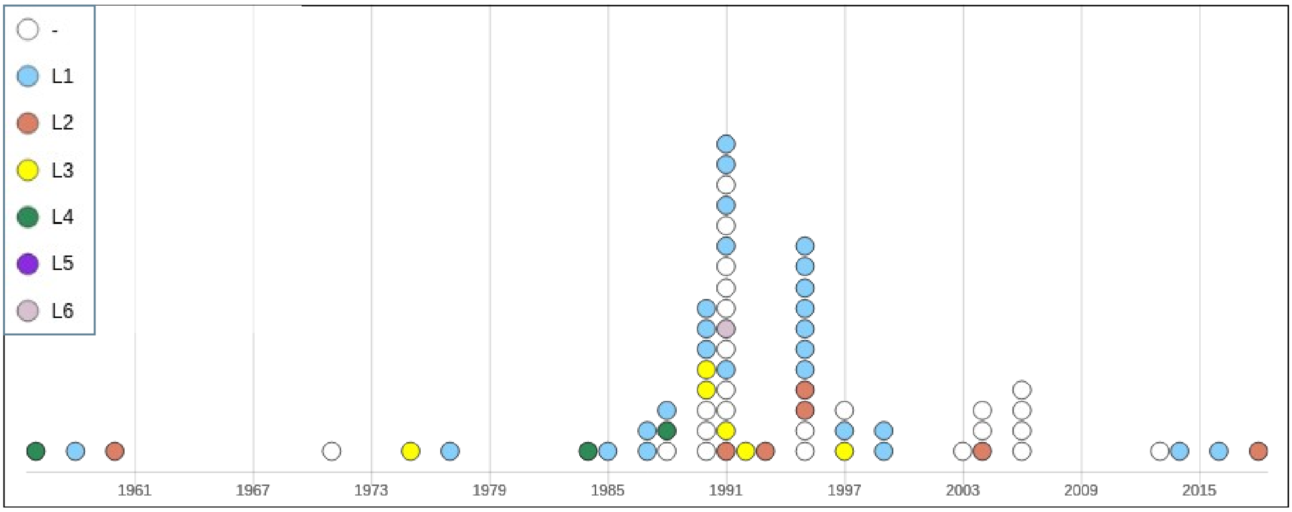

Figure S6: Timeline of WGS isolate sampling. As not all isolates used have known sampling times this represents 67 of the 105 public and newly sequenced isolates used. Isolates are coloured by major lineage.

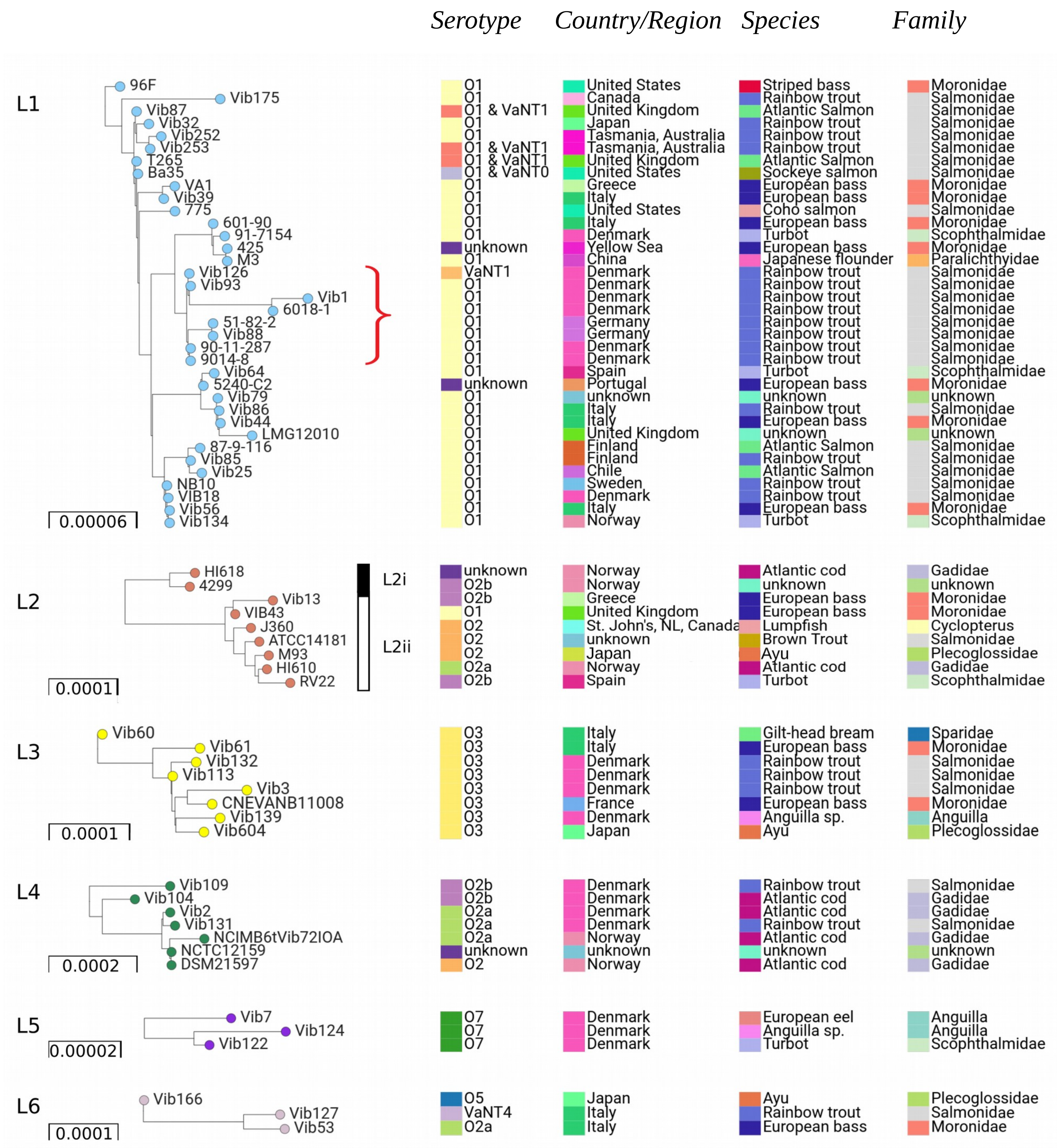

Figure S7: Subtrees of six major lineages (post-recombination removal) with corresponding metadata. The curly red bracket indicates a L1 sub-lineage potentially associated with Rainbow trout. The two lineages of L2 (L2i and L2ii) that are highly distinctive by the MLSA data are shown by the black and white bars respectively.

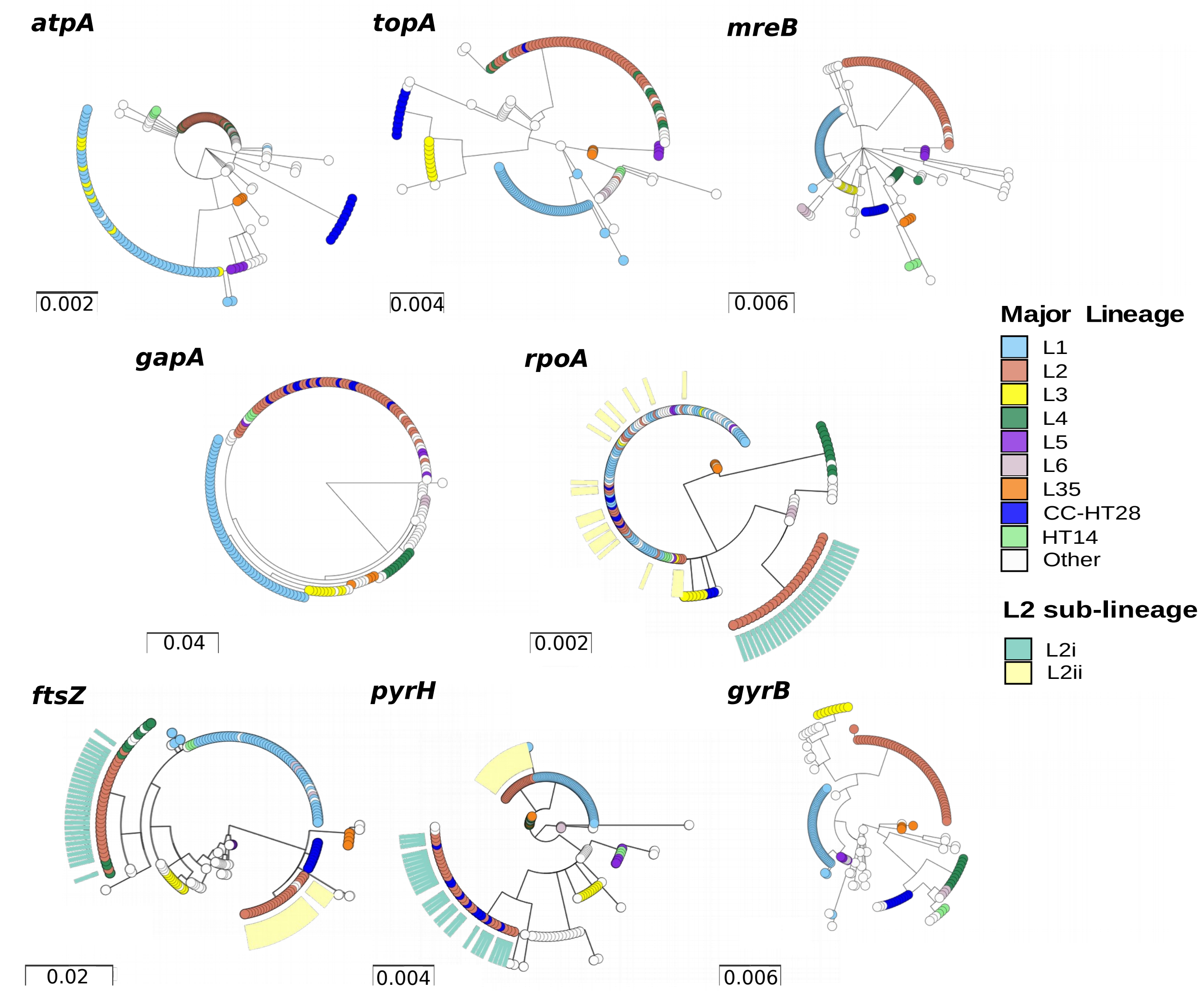

Figure S8: Individual trees for eight MLSA loci constructed using FASTtree. Leaves are coloured by major lineage. All isolates not included in these major lineages are coloured white. Sub-lineages L2i and L2ii are highlighted in loci *rpoA*, *ftsZ* and *pyrH*.

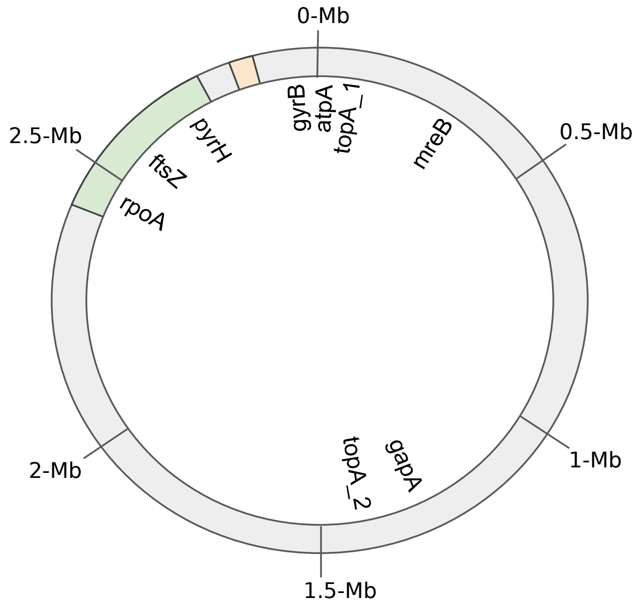

Figure S9: Illustration of positions of MLSA loci on *Vibrio anguillarum* VIB43 chromosome 1. The positions of the large homologous recombination region (green) and the LPS biosynthesis related genomic island (beige) are also indicated. Loci *pyrH*, *ftsZ* and *rpoA* are located within the large homologous recombination.

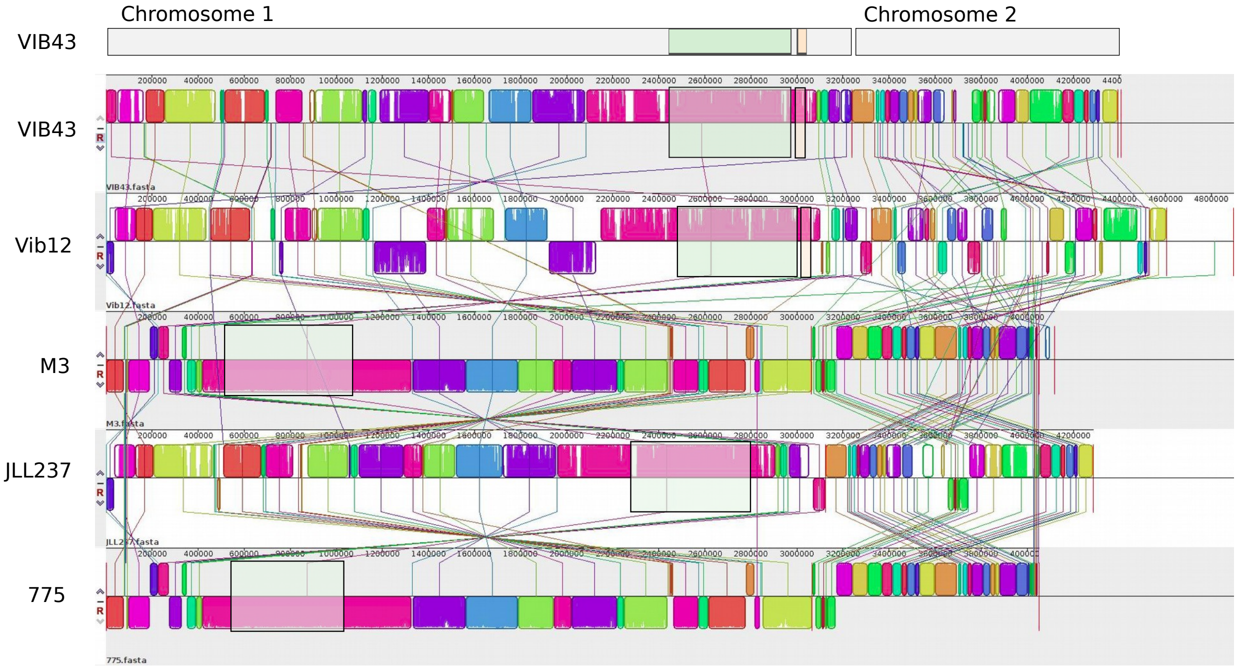

Figure S10: ProgressiveMauve alignment of five complete genomes reveal that the large homologous replacement occurs on a long Locally Collinear Block (LCB). Isolates included are VIB43 (L2i), VIB12 (L27), M3 (L1), JLL237 (L8), and ATCC 68554 (775 - L1). The positions of the 0.5-Mb homologous recombination and the LPS biosynthesis related genomic island as identified in VIB43 are indicated in green and beige respectively. Both recombination events are located on a large pink Locally Collinear Block (LCB), which is a region that does not contain genome rearrangements between isolates.

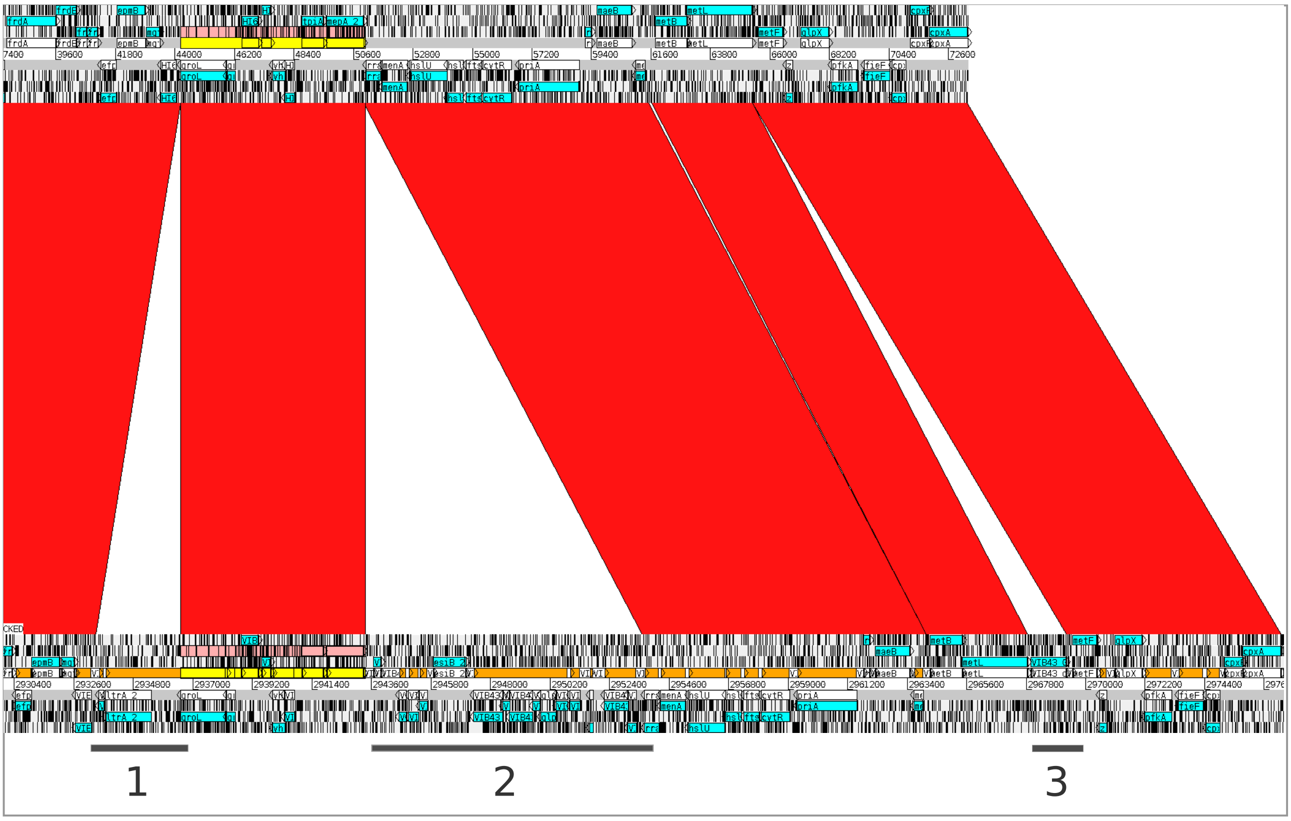

Figure S11: Genes within the large homologous recombination absent in L2i. ACT comparison of seqeunces within HI618 (L2i - top) and VIB43 (L2ii – bottom). Three labelled segments in VIB43 represent genes found in L2ii isolates but missing in L2i isolates. Segment 2 consists of a group II intron reverse transcriptase/maturase and a DUF1508 containing protein. Segment 2 contains 15 genes including six transposases, an integrase core domain protein, *luxR*, an SLR family protein, and a rhomboid family protease. The third segment contains one gene, a transposase.

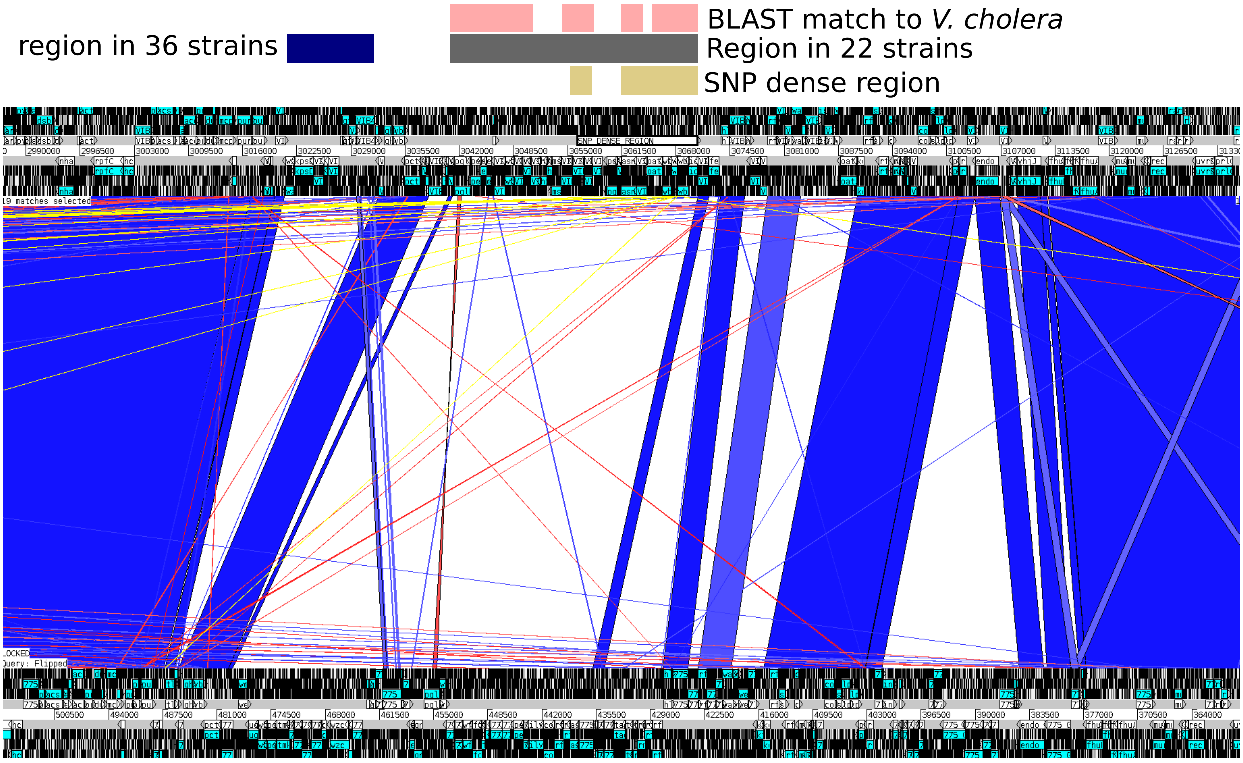

Figure S12: Artemis Comparison Tool comparison of L2ii strain VIB43 and L1 strain 775, reveals variation in LPS biosynthesis related region of chromosome 1. VIB43 and 775 contain different sets of genes in a region. The gene set found in VIB43 is shared with 22 isolates. All known serotype 02 strains are included in this set of isolates. Some of this region in VIB43 shows similarity to a LPS-biosynthesis related region in *V. cholera* (Aydanian et al. 2011) (GU576499.1*)*. Another smaller region missing in 775 and exclusive to 36 isolates, contains capsule-related genes.

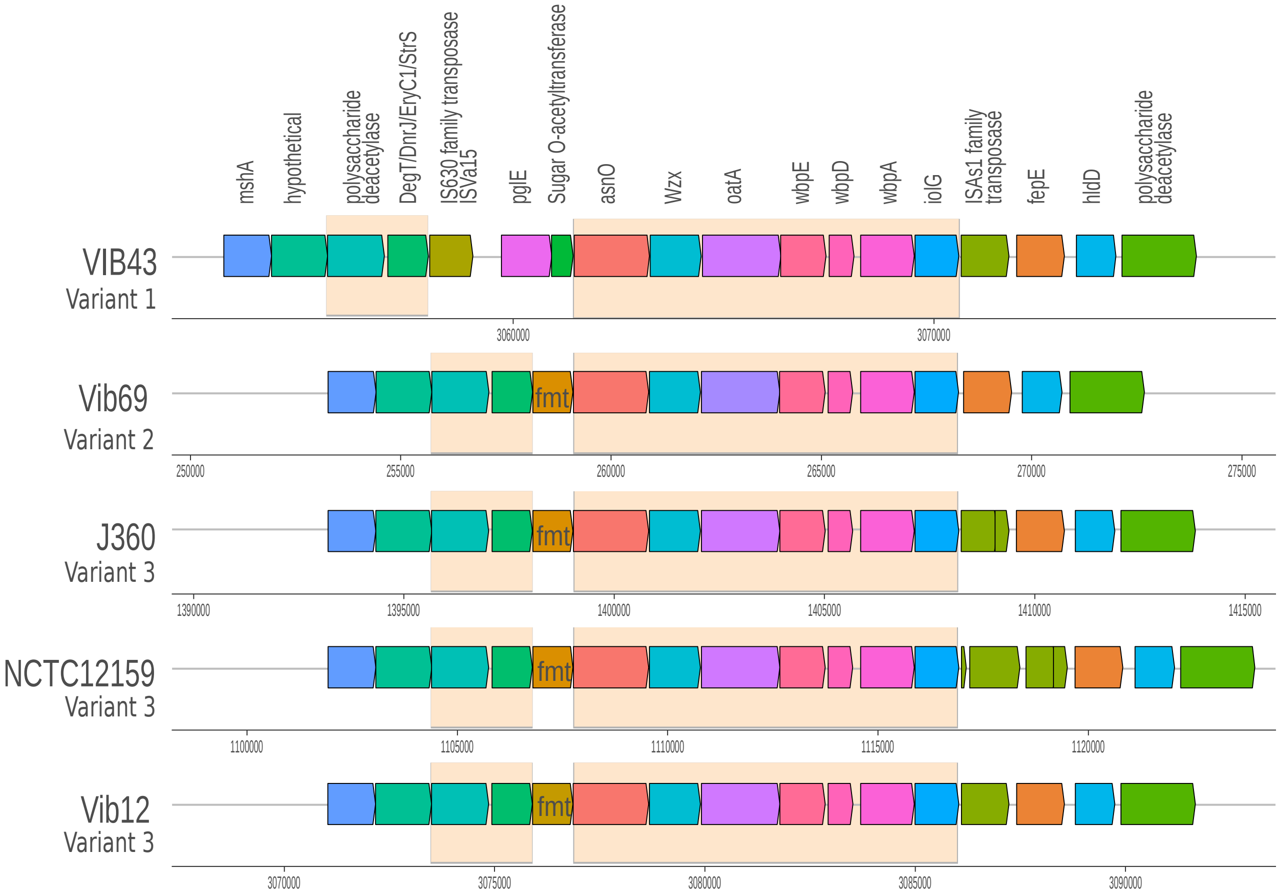

Figure S13: Comparison of gene content on and beside the SNP dense region of the LPS biosynthesis-related genomic island in five representative isolates. Genes with high nucleotide variation between isolates are shown in beige. VIB43 (L2ii) contains variant 1 of the GI, which differs from the rest of L2ii and is instead similar to L2i. It also contains a variation in gene content where *fmt* has been replaced by three genes - a ISVa15 transposase, *pglE,* and a sugar O-acetyltransferase. L2i isolates and Vib13 appear to have the same variant of this recombination region as VIB43 (variant 1). Vib69 represents variant 2 of this GI, also found in Vib552 and Vib77. These isolates do not contain a copy of the ISAs1 family transposase found in all other isolates. J360 represents L2ii and contains two copies of the ISAs1 family transposase. NCT 12159 represents L4 isolates and contains five copies of the ISAs1 family transposase. VIB12 (L27) contains a similar variant in both nucleotide identity and gene content to L2ii isolates. Contig breaks around this region are found in many of the other isolates, possibly due to the presence of transposases.

Supplementary note:

A gene cluster highlighted in blue in Figure S8, is consistently observed in 36 of the 105 isolates examined. Isolates carrying variants 1 and 3 of the previous gene cluster all carry this second set of genes. The three isolates containing variant 2 of the previous gene cluster do not. In addition to these largely O2 related genomes, we can identify this gene set in all isolates with serotypes O3 and O7. A phylogenetic analysis of this cluster, as seen in Figure S10, separates isolates carrying the larger gene cluster, from all other isolates. Serotype O3 and O7 strains from Lineages L3 and L5 respectively each contain distinct highly conserved variants of this region. Serotype O7 strains not found within L5 (Vib608 and Vib54) contain a different variant of this region. Similarly, serotype O3 strains not included in L3 don’t share the same variant of this region (PF7, PF430-3 and PF4). This clear separation of serotypes O2, O3, and O7 indicates that genes found in this block are necessary for forming these distinct serotypes.

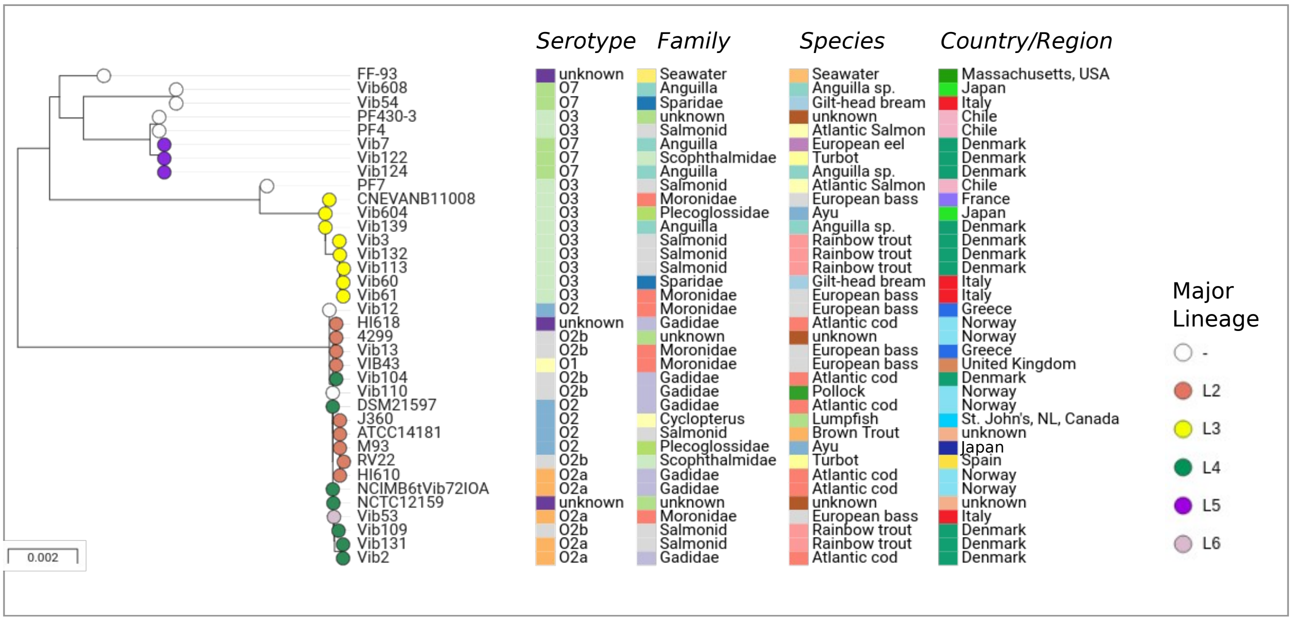

Figure S14: Tree produced using LPS and capsule related genomic island for the 36 isolates that carry this accessory region. The tree was built using SNPs called against this segment (11,056-bp) in VIB43. The tree groups all O2 associated isolates and L2i isolate VIB43 as one cluster, indicating they all share the same variant of this gene cluster. Serotype O3 isolates from major lineage L3 are generally homogenous in this region. Similarly all isolates from L5 share a highly similar variant of this gene set. Other isolates possessing this region of genes, not found within these major lineages, consist of all other isolates in the dataset that have been classified as either serotype O3 and O7. These isolates do not group with their assigned serotype with respect to this region.

Table S1: Metadata for all 189 strains

Isolate id (other names)	Data Type	Data Source	Accession	Major Lineage	ST	Species Designation	Source of isolation (Family)	Country/Region	Year	Serogroup
**Vib552 (UB V078)**	WGS	This Publication (Austin et al 1997)		Other	Unknown	V. anguillarum	Gilt-head bream (Sparidae)	Spain	1991	O2b
**Vib553 (UB A085)**	WGS	This Publication (Austin et al 1997)		Other	Unknown	V. anguillarum	Rotifer (Rotifer)	Spain	1991	O6
**Vib604 (UB (PT-493))**	WGS	This Publication (Austin et al 1997)		L3	Unknown	V. anguillarum	Ayu (Plecoglossidae)	Japan	1975	O3
**Vib608 (UB (ET-1))**	WGS	This Publication (Austin et al 1997)		Other	Unknown	V. anguillarum	Anguilla sp. (Anguilla)	Japan	1971	O7
**NCIMB6tVib72IOA (LMG 4437; NCIMB 6T; Vib72)**	WGS & MLSA	This Publication (Austin et al 1995)		L4	HT_1	V. anguillarum	Atlantic cod (Gadidae)	Norway	1956	O2a
**Vib1 (ATCC 43305)**	WGS & MLSA	This Publication (Austin et al 1995)		L1	HT_4	V. anguillarum	Rainbow trout (Salmonid)	Denmark	unknown	O1
**Vib10 (ATCC 43314)**	WGS & MLSA	This Publication (Austin et al 1995)		Other	HT_12	V. anguillarum	Atlantic cod (Gadidae)	Denmark	unknown	10
**Vib104 (RVAU 1474/1)**	WGS & MLSA	This Publication (Austin et al 1995)		L4	HT_1	V. anguillarum	Atlantic cod (Gadidae)	Denmark	unknown	O2b
**Vib127 (RVAU 3737/1)**	WGS & MLSA	This Publication (Austin et al 1995)		L6	HT_35	V. anguillarum	Rainbow trout (Salmonid)	Italy	unknown	VaNT4
**Vib253 (Carson 85/3954-4)**	WGS & MLSA	This Publication (Austin et al 1995)		L1	HT_37	V. anguillarum	Rainbow trout (Salmonid)	Tasmania, Australia	unknown	O1
**Vib4 (ATCC 43308)**	WGS & MLSA	This Publication (Austin et al 1995)		Other	HT_6	V. anguillarum	Atlantic cod (Gadidae)	Denmark	unknown	O4
**Vib5 (ATCC 43309)**	WGS & MLSA	This Publication (Austin et al 1995)		Other	HT_7	V. anguillarum	Atlantic cod (Gadidae)	Denmark	unknown	O5
**Vib6 (ATCC 43310)**	WGS & MLSA	This Publication (Austin et al 1995)		Other	Unknown	V. anguillarum	Atlantic cod (Gadidae)	Denmark	unknown	O6
**Vib64 (UB A023)**	WGS & MLSA	This Publication (Austin et al 1995)	GCA_001989675.1 ***	L1	HT_4	V. anguillarum	Turbot (Scophthalmidae)	Spain	1988; [1995]	O1
**Vib69 (UB A078)**	WGS & MLSA	This Publication (Austin et al 1995)		Other	Unknown	V. anguillarum	Gilt-head bream (Sparidae)	Spain	1991	O2a
**Vib77 (LMG 12099)**	WGS & MLSA	This Publication (Austin et al 1995)		Other	HT_38	V. anguillarum	Ayu (Plecoglossidae)	Japan	unknown	O2a;O2b
**Vib8 (ATCC 43312)**	WGS & MLSA	This Publication (Austin et al 1995)		Other	HT_10	V. anguillarum	Atlantic cod (Gadidae)	Denmark	unknown	O8
**Vib87 (NCMB 1873)**	WGS & MLSA	This Publication (Austin et al 1995)		L1	HT_4	V. anguillarum	Atlantic Salmon (Salmonid)	United Kingdom	unknown	O1 & VaNT1
**Vib9 (ATCC 43313)**	WGS & MLSA	This Publication (Austin et al 1995)		Other	HT_11	V. anguillarum	Atlantic cod (Gadidae)	Denmark	unknown	O9
**Vib109 (RVAU 860828-1/3a)**	WGS	This Publication (Austin et al 1995)		L4	Unknown	V. anguillarum	Rainbow trout (Salmonid)	Denmark	unknown	O2b
**Vib110 (RVAU 90-2-6)**	WGS	This Publication (Austin et al 1995)		Other	Unknown	V. anguillarum	Pollock (Gadidae)	Norway	unknown	O2b
**Vib113 (RVAU 820806-1/3)**	WGS	This Publication (Austin et al 1995)		L3	Unknown	V. anguillarum	Rainbow trout (Salmonid)	Denmark	unknown	O3
**Vib114 (RVAU 1712/1)**	WGS	This Publication (Austin et al 1995)		Other	Unknown	V. anguillarum	Atlantic cod (Gadidae)	Denmark	unknown	O4
**Vib115 (RVAU 74/82/1)**	WGS	This Publication (Austin et al 1995)		Other	Unknown	V. anguillarum	Rainbow trout (Salmonid)	Germany	unknown	O4
**Vib117 (RVAU 89-9-181)**	WGS	This Publication (Austin et al 1995)		Other	Unknown	V. anguillarum	Atlantic Salmon (Salmonid)	Norway	unknown	O4
**Vib118 (RVAU 1629)**	WGS	This Publication (Austin et al 1995)		Other	Unknown	V. anguillarum	Atlantic cod (Gadidae)	Denmark	unknown	O5
**Vib119 (RVAU 850816-1/17b)**	WGS	This Publication (Austin et al 1995)		Other	Unknown	V. anguillarum	Rainbow trout (Salmonid)	Denmark	unknown	O5
**Vib122 (RVAU 6828g)**	WGS	This Publication (Austin et al 1995)		L5	HT_9	V. anguillarum	Turbot (Scophthalmidae)	Denmark	unknown	O7
**Vib124 (RVAU 1702/1)**	WGS	This Publication (Austin et al 1995)		L5	HT_9	V. anguillarum	Anguilla sp. (Anguilla)	Denmark	unknown	O7
**Vib125 (RVAU 88-12-716)**	WGS	This Publication (Austin et al 1995)		Other	Unknown	V. anguillarum	Atlantic Salmon (Salmonid)	Norway	1988	O8
**Vib126 (RVAU 840606-2/5)**	WGS	This Publication (Austin et al 1995)		L1	HT_4	V. anguillarum	Rainbow trout (Salmonid)	Denmark	unknown	VaNT1
**Vib128 (RVAU 850610-1/8b)**	WGS	This Publication (Austin et al 1995)		Other	Unknown	V. anguillarum	Rainbow trout (Salmonid)	Denmark	unknown	VaNT2
**Vib13 (AVL 68-28-C)**	WGS	This Publication (Austin et al 1995)		L2	HT_3	V. anguillarum	European bass (Moronidae)	Greece	1991	O2b
**Vib131 (RVAU 88-6-73/2)**	WGS	This Publication (Austin et al 1995)		L4	HT_1	V. anguillarum	Rainbow trout (Salmonid)	Denmark	1988	O2a
**Vib132 (RVAU 91-7-143)**	WGS	This Publication (Austin et al 1995)		L3	Unknown	V. anguillarum	Rainbow trout (Salmonid)	Denmark	1991	O3

**Vib139 (RVAU 92-8-168)**	WGS	This Publication (Austin et al 1995)		L3	Unknown	V. anguillarum	Anguilla sp. (Anguilla)	Denmark	1992	O3
**Vib166 (RVAU PB15)**	WGS	This Publication (Austin et al 1995)		L6	HT_35	V. anguillarum	Ayu (Plecoglossidae)	Japan	unknown	O5
**Vib175 (RVAU VA12)**	WGS	This Publication (Austin et al 1995)		L1	HT_4	V. anguillarum	Rainbow trout (Salmonid)	Canada	unknown	O1
**Vib2 (ATCC 43306)**	WGS	This Publication (Austin et al 1995)		L4	HT_1	V. anguillarum	Atlantic cod (Gadidae)	Denmark	unknown	O2a
**Vib203 (LMG 13230)**	WGS	This Publication (Austin et al 1995)		Other	Unknown	V. anguillarum	Gilt-head bream (Sparidae)	Spain	1991	O6
**Vib222 (LMG 13249)**	WGS	This Publication (Austin et al 1995)		Other	Unknown	V. anguillarum	European bass (Moronidae)	Spain	1991	O6
**Vib25 (HWU 63)**	WGS	This Publication (Austin et al 1995)		L1	HT_4	V. anguillarum	Atlantic Salmon (Salmonid)	Chile	1991	O1
**Vib252 (Carson 86/3831-2)**	WGS	This Publication (Austin et al 1995)		L1	HT_4	V. anguillarum	Rainbow trout (Salmonid)	Tasmania, Australia	unknown	O1
**Vib3 (ATCC 43307)**	WGS	This Publication (Austin et al 1995)		L3	HT_5	V. anguillarum	Rainbow trout (Salmonid)	Denmark	unknown	O3
**Vib32 (NCMB 572)**	WGS	This Publication (Austin et al 1995)		L1	HT_4	V. anguillarum	Rainbow trout (Salmonid)	Japan	1958	O1
**Vib37 (UB 109/90)**	WGS	This Publication (Austin et al 1995)		Other	HT_6	V. anguillarum	Gilt-head bream (Sparidae)	Italy	1990	O4
**Vib41 (UB 207/90)**	WGS	This Publication (Austin et al 1995)		Other	Unknown	V. anguillarum	Gilt-head bream (Sparidae)	Italy	1990	O8
**Vib45 (UB 295/91)**	WGS	This Publication (Austin et al 1995)		Other	Unknown	V. anguillarum	European bass (Moronidae)	Italy	1991	O4
**Vib53 (UB 258/91)**	WGS	This Publication (Austin et al 1995)		L6	Unknown	V. anguillarum	European bass (Moronidae)	Italy	1991	O2a
**Vib54 (UB 574/91)**	WGS	This Publication (Austin et al 1995)		Other	Unknown	V. anguillarum	Gilt-head bream (Sparidae)	Italy	1991	O7
**Vib56 (UB 601/91)**	WGS	This Publication (Austin et al 1995)		L1	HT_4	V. anguillarum	European bass (Moronidae)	Italy	1991	O1
**Vib60 (UB 910/90)**	WGS	This Publication (Austin et al 1995)		L3	Unknown	V. anguillarum	Gilt-head bream (Sparidae)	Italy	1990	O3
**Vib61 (UB 960/90)**	WGS	This Publication (Austin et al 1995)		L3	Unknown	V. anguillarum	European bass (Moronidae)	Italy	1990	O3
**Vib7 (ATCC 43311)**	WGS	This Publication (Austin et al 1995)		L5	HT_9	V. anguillarum	European eel (Anguilla)	Denmark	unknown	O7
**Vib79 (LMG 12101)**	WGS	This Publication (Austin et al 1995)		L1	HT_4	V. anguillarum	unknown (unknown)	unknown	1991	O1
**Vib82 (LMG 13186)**	WGS	This Publication (Austin et al 1995)		L35	HT_20	V. anguillarum	European bass (Moronidae)	Greece	1991	VaNT2
**Vib86 (RVAU 1692)**	WGS	This Publication (Austin et al 1995)		L1	HT_4	V. anguillarum	Rainbow trout (Salmonid)	Italy	unknown	O1
**Vib88 (RVAU 51/82/2)**	WGS	This Publication (Austin et al 1995)		L1	HT_4	V. anguillarum	Rainbow trout (Salmonid)	Germany	unknown	O1
**Vib134 (RVAU 91-8-178)**	WGS	This Publication (Austin et al 1995,Castillo et al 2017)	GCA_001989735.1 ***	L1	HT_4	V. anguillarum	Turbot (Scophthalmidae)	Norway	1991	O1
**Vib39 (UB 178/90)**	WGS	This Publication (Austin et al 1995,Castillo et al 2017)	GCA_001989755.1 ***	L1	HT_4	V. anguillarum	European bass (Moronidae)	Italy	1990	O1
**Vib44 (UB 261/91; 261/91)**	WGS	This Publication (Austin et al 1995,Castillo et al 2017)	GCA_001989775.1 ***	L1	HT_4	V. anguillarum	European bass (Moronidae)	Italy	1991; [1995]	O1
**Vib85 (RVAU 87-9-117; 87-9-117)**	WGS	This Publication (Austin et al 1995,Castillo et al 2017)	GCA_001989715.1 ***	L1	HT_4	V. anguillarum	Rainbow trout (Salmonid)	Finland	1987	O1
**Vib93 (RVAU 850610-1/6a)**	WGS	This Publication (Austin et al 1995,Castillo et al 2017)	GCA_001990125.1 ***	L1	HT_4	V. anguillarum	Rainbow trout (Salmonid)	Denmark	1985	O1
**5240-C2**	WGS	This Publication		L1	HT_4	V. anguillarum	European bass (Moronidae)	Portugal	2016	unknown
**HI618**	WGS	Public (Rønneseth et al 2017)	GCA_002078035.1	L2	HT_2	V. anguillarum	Atlantic cod (Gadidae)	Norway	1995	unknown
**90-11-286**	WGS (Complete genome)	Public (Rasmussen et al. 2016)	GCA_001660505.1	Other	Unknown	V. anguillarum	Rainbow trout (Salmonid)	Denmark	1990	O1
**425**	WGS (Complete genome)	Public (NCBI)	GCA_003031205.1	L1	HT_4	V. anguillarum	European bass (Moronidae)	Yellow Sea	1999	unknown
**J360 (VaJ360)**	WGS	Public (NCBI)	GCA_003399575.1	L2	HT_3	V. anguillarum	Lumpfish (Cyclopterus)	St. John's, NL, Canada	2018	O2
**MHK3**	WGS (Complete genome)	Public (NCBI)	GCA_003595585.1	Other	HT_38	V. anguillarum	Flounder (Paralichthyidae)	Weihai, Shandong, China	2006	unknown
**ATCC14181**	WGS	Public (NCBI)	GCA_001718015.1	L2	HT_3	V. anguillarum	Brown Trout (Salmonid)	unknown	1960	O2
**LMG12010**	WGS	Public (NCBI)	GCA_001989875.1	L1	HT_4	V. anguillarum	unknown (unknown)	United Kingdom	1995	O1

**M93**	WGS	Public (NCBI)	GCA_002901125.1	L2	HT_3	V. anguillarum	Ayu (Plecoglossidae)	Lake Biwa, Shiga Prefecture, Japan	1993	O2
**NCTC12159 (ERS1403243)**	WGS	Public (NCBI)	GCA_900452855.1	L4	HT_1	V. anguillarum	unknown (unknown)	unknown	unknown	unknown
**775 (ATCC 68554, VIB169)**	WGS & MLSA (Complete genome)	Public (Naka et al. 2011)	GCA_000217675.1	L1	HT_39	V. anguillarum	Coho salmon (Salmonid)	United States	1977	O1
**96F**	WGS & MLSA	Public (Naka et al. 2011)	GCA_000257165.1	L1	HT_40	V. anguillarum	Striped bass (Moronidae)	United States	unknown	O1
**RV22**	WGS & MLSA	Public (Naka et al. 2011)	GCA_000257185.1	L2	HT_3	V. anguillarum	Turbot (Scophthalmidae)	Spain	unknown	O2b
**M3**	WGS & MLSA (Complete genome)	Public (Li et al. 2013)	GCA_000462975.1	L1	HT_4	V. anguillarum	Japanese flounder (Paralichthyidae)	China	1999	O1
**CNEVANB11008**	WGS (Complete genome)	Public (Holm et al. 2018)	GCA_002212025.1	L3	Unknown	V. anguillarum	European bass (Moronidae)	France	1997	O3
**JLL237**	WGS (Complete genome)	Public (Holm et al. 2018)	GCA_002211985.1	Other	Unknown	V. anguillarum	Rainbow trout (Salmonid)	Denmark	1995	O1
**Vib12 (89-2-54)**	WGS (Complete genome)	Public (Holm et al. 2018)	GCA_002310335.1	Other	Unknown	V. anguillarum	European bass (Moronidae)	Greece	unknown	O2
**VIB43**	WGS (Complete genome)	Public (Holm et al. 2018)	GCA_002287545.1	L2	HT_3	V. anguillarum	European bass (Moronidae)	United Kingdom	unknown	O1
**87-9-116**	WGS	Public (Holm et al. 2018)	GCA_002211505.1	L1	HT_4	V. anguillarum	Atlantic Salmon (Salmonid)	Finland	1987	O1
**NB10**	WGS & MLSA (Complete genome)	Public (Holm et al. 2015)	GCA_000786425.1	L1	HT_4	V. anguillarum	Rainbow trout (Salmonid)	Sweden	unknown	O1
**12B09**	WGS	Public (Cordero et al 2012)	GCA_000287135.2	Other	Unknown	V. anguillarum**	Seawater (Seawater)	Plum Island, Norway	2003	unknown
**FF-93**	WGS	Public (Cordero et al 2012)	GCA_000287095.2	Other	Unknown	V. anguillarum**	Seawater (Seawater)	Norway	2006	unknown
**FS-144**	WGS	Public (Cordero et al 2012)	GCA_000287115.2	Other	Unknown	V. anguillarum**	Seawater (Seawater)	Norway	2006	unknown
**FS-238**	WGS	Public (Cordero et al 2012)	GCA_000287155.2	Other	Unknown	V. anguillarum**	Seawater (Seawater)	Norway	2006	unknown
**4299**	WGS & MLSA	Public (Castillo et al 2017)	GCA_001989655.1	L2	HT_2	V. anguillarum	unknown (unknown)	Norway	1995	O2b
**S3-4-9**	WGS (Complete genome)	Public (Castillo et al 2017)	GCA_002212005.1	Other	Unknown	V. anguillarum	Rainbow trout (Salmonid)	Denmark	1995	O1
**51-82-2**	WGS	Public (Castillo et al 2017)	GCA_001989855.1	L1	HT_4	V. anguillarum	Rainbow trout (Salmonid)	Germany	1997	O1
**601-90**	WGS	Public (Castillo et al 2017)	GCA_001990065.1	L1	HT_4	V. anguillarum	European bass (Moronidae)	Italy	1995	O1
**6018-1**	WGS	Public (Castillo et al 2017)	GCA_001990085.1	L1	HT_4	V. anguillarum	Rainbow trout (Salmonid)	Denmark	1995	O1
**90-11-287**	WGS	Public (Castillo et al 2017)	GCA_001990025.1	L1	HT_4	V. anguillarum	Rainbow trout (Salmonid)	Denmark	1990	O1
**9014-8**	WGS	Public (Castillo et al 2017)	GCA_001989915.1	L1	HT_4	V. anguillarum	Rainbow trout (Salmonid)	Denmark	1990	O1
**91-7154 (Unnamed on NCBI)**	WGS	Public (Castillo et al 2017)	GCA_001990045.1	L1	HT_4	V. anguillarum	Turbot (Scophthalmidae)	Denmark	1995	O1
**Ba35**	WGS	Public (Castillo et al 2017)	GCA_001989795.1	L1	HT_4	V. anguillarum	Sockeye salmon (Salmonid)	United States	1995	O1 & VaNT0
**DSM21597**	WGS	Public (Castillo et al 2017)	GCA_001989995.1	L4	HT_1	V. anguillarum	Atlantic cod (Gadidae)	Norway	1984	O2
**HI610**	WGS	Public (Castillo et al 2017)	GCA_001989835.1	L2	HT_3	V. anguillarum	Atlantic cod (Gadidae)	Norway	2004	O2a
**PF4**	WGS	Public (Castillo et al 2017)	GCA_003595565.1	Other	Unknown	V. anguillarum	Atlantic Salmon (Salmonid)	Chile	2004	O3
**PF430-3**	WGS	Public (Castillo et al 2017)	GCA_001989695.1	Other	Unknown	V. anguillarum	unknown (unknown)	Chile	2013	O3
**PF7**	WGS	Public (Castillo et al 2017)	GCA_001997225.1	Other	Unknown	V. anguillarum	Atlantic Salmon (Salmonid)	Chile	2004	O3
**S22-9**	WGS	Public (Castillo et al 2017)	GCA_001989895.1	Other	Unknown	V. anguillarum	Rainbow trout (Salmonid)	Denmark	1997;	O1
**T265**	WGS	Public (Castillo et al 2017)	GCA_001989815.1	L1	HT_4	V. anguillarum	Atlantic Salmon (Salmonid)	United Kingdom	1995	O1 & VaNT1

**VA1**	WGS	Public (Castillo et al 2017)	GCA_001990105.1	L1	HT_4	V. anguillarum	European bass (Moronidae)	Greece	2014	O1
**VIB18 (AVL 27.2)**	WGS	Public (Castillo et al 2017)	GCA_001998845.1	L1	HT_4	V. anguillarum	Rainbow trout (Salmonid)	Denmark	1995	O1
**820723**	MLSA	MLSA (Steinum et al. 2016)		L4	HT_1	V. anguillarum	unknown (unknown)	Denmark	unknown	O2b
**1173_1 (Vib 88)**	MLSA	MLSA (Steinum et al. 2016)		L4	HT_1	V. anguillarum	Atlantic cod (Gadidae)	Denmark	unknown	unknown
**774_02**	MLSA	MLSA (Steinum et al. 2016)		L2	HT_2	V. anguillarum	Atlantic cod (Gadidae)	Scotland	unknown	unknown
**860709_6_1**	MLSA	MLSA (Steinum et al. 2016)		L2	HT_3	V. anguillarum	Rainbow trout (Salmonid)	Denmark	unknown	O2c
**860813_17_1b**	MLSA	MLSA (Steinum et al. 2016)		L2	HT_3	V. anguillarum	Rainbow trout (Salmonid)	Denmark	unknown	O2c
**910614_1_1**	MLSA	MLSA (Steinum et al. 2016)		L2	HT_3	V. anguillarum	Atlantic cod (Gadidae)	Denmark	unknown	O2c
**ATCC43306**	MLSA	MLSA (Steinum et al. 2016)		L4	HT_1	V. anguillarum	Atlantic cod (Gadidae)	Denmark	unknown	O2a
**ATCC43307**	MLSA	MLSA (Steinum et al. 2016)		L3	HT_5	V. anguillarum	Rainbow trout (Salmonid)	Denmark	unknown	O3
**ATCC43311**	MLSA	MLSA (Steinum et al. 2016)		L5	HT_9	V. anguillarum	European eel (Anguilla)	Denmark	unknown	O7
**HI21412**	MLSA	MLSA (Steinum et al. 2016)		Other	HT_13	V. anguillarum	Atlantic cod (Gadidae)	Norway	unknown	unknown
**HI21413**	MLSA	MLSA (Steinum et al. 2016)		L45	HT_14	V. anguillarum	Atlantic cod (Gadidae)	Norway	unknown	unknown
**HI21414**	MLSA	MLSA (Steinum et al. 2016)		L45	HT_14	V. anguillarum	Atlantic cod (Gadidae)	Norway	unknown	unknown
**HI21429**	MLSA	MLSA (Steinum et al. 2016)		L45	HT_14	V. anguillarum	Atlantic cod (Gadidae)	Norway	unknown	unknown
**IU_01**	MLSA	MLSA (Steinum et al. 2016)		L1	HT_15	V. anguillarum	European bass (Moronidae)	Turkey	unknown	non-O1/O2
**IU_02**	MLSA	MLSA (Steinum et al. 2016)		L1	HT_16	V. anguillarum	European bass (Moronidae)	Turkey	unknown	non-O1/O2
**IU_03**	MLSA	MLSA (Steinum et al. 2016)		L2	HT_3	V. anguillarum	European bass (Moronidae)	Turkey	unknown	non-O1/O2
**IU_04**	MLSA	MLSA (Steinum et al. 2016)		L1	HT_4	V. anguillarum	Flathead grey mullet (Mugilidae)	Turkey	unknown	O1
**IU_05**	MLSA	MLSA (Steinum et al. 2016)		L1	HT_4	V. anguillarum	European bass (Moronidae)	Turkey	unknown	non-O1/O2
**IU_07**	MLSA	MLSA (Steinum et al. 2016)		L1	HT_17	V. anguillarum	European bass (Moronidae)	Turkey	unknown	non-O1/O2
**IU_08**	MLSA	MLSA (Steinum et al. 2016)		L1	HT_4	V. anguillarum	European bass (Moronidae)	Turkey	unknown	non-O1/O2
**IU_10**	MLSA	MLSA (Steinum et al. 2016)		L1	HT_18	V. anguillarum	European bass (Moronidae)	Turkey	unknown	non-O1/O2
**IU_11**	MLSA	MLSA (Steinum et al. 2016)		L1	HT_4	V. anguillarum	Atlantic bluefin tuna (Scombridae)	Turkey	unknown	non-O1/O2
**IU_12**	MLSA	MLSA (Steinum et al. 2016)		L1	HT_19	V. anguillarum	European bass (Moronidae)	Turkey	unknown	non-O1/O2
**LMG13224**	MLSA	MLSA (Steinum et al. 2016)		L35	HT_20	V. anguillarum	Rotifer (Rotifer)	Greece	unknown	non-O1/O2
**LMG13225**	MLSA	MLSA (Steinum et al. 2016)		L35	HT_20	V. anguillarum	Rotifer (Rotifer)	Greece	unknown	non-O1/O2
**LMG13227**	MLSA	MLSA (Steinum et al. 2016)		L35	HT_21	V. anguillarum	Gilt-head bream (Sparidae)	Greece	unknown	non-O1/O2
**N3A3_2**	MLSA	MLSA (Steinum et al. 2016)		Other	HT_22	V. anguillarum	Atlantic cod (Gadidae)	Scotland	unknown	non-O1/O2
**N8D10_8**	MLSA	MLSA (Steinum et al. 2016)		Other	HT_22	V. anguillarum	Atlantic cod (Gadidae)	Scotland	unknown	unknown
**NCIMB1873**	MLSA	MLSA (Steinum et al. 2016)		L1	HT_4	V. anguillarum	Chinook salmon (Salmonid)	United States	unknown	O1
**NCIMB2129**	MLSA	MLSA (Steinum et al. 2016)		L1	HT_4	V. anguillarum	Rainbow trout (Salmonid)	Norway	unknown	O1
**NCIMB2130**	MLSA	MLSA (Steinum et al. 2016)		L2	HT_23	V. anguillarum	Pollock (Gadidae)	Norway	unknown	O2b
**NCIMB2131**	MLSA	MLSA (Steinum et al. 2016)		L1	HT_4	V. anguillarum	Rainbow trout (Salmonid)	Norway	unknown	O1
**NCIMB2132**	MLSA	MLSA (Steinum et al. 2016)		L1	HT_4	V. anguillarum	Rainbow trout (Salmonid)	Norway	unknown	O1
**NCIMB2133**	MLSA	MLSA (Steinum et al. 2016)		L2	HT_2	V. anguillarum	Pollock (Gadidae)	Norway	unknown	O2b
**NVI3379**	MLSA	MLSA (Steinum et al. 2016)		L2	HT_3	V. anguillarum	Turbot (Scophthalmidae)	Norway	unknown	O2a
**NVI4299**	MLSA	MLSA (Steinum et al. 2016)		L2	HT_2	V. anguillarum	Atlantic cod (Gadidae)	Norway	unknown	O2b
**NVI4317**	MLSA	MLSA (Steinum et al. 2016)		L1	HT_26	V. anguillarum	Rainbow trout (Salmonid)	Norway	unknown	O1
**NVI4351**	MLSA	MLSA (Steinum et al. 2016)		L41	HT_27	V. anguillarum	Atlantic cod (Gadidae)	Norway	unknown	O2a biotype II
**NVI4353**	MLSA	MLSA (Steinum et al. 2016)		L2	HT_2	V. anguillarum	Atlantic cod (Gadidae)	Norway	unknown	O2b
**NVI4590**	MLSA	MLSA (Steinum et al. 2016)		L2	HT_2	V. anguillarum	Atlantic cod (Gadidae)	Norway	unknown	O2b
**NVI4591**	MLSA	MLSA (Steinum et al. 2016)		L2	HT_3	V. anguillarum	Atlantic Salmon (Salmonid)	Norway	unknown	O2a
**NVI4601**	MLSA	MLSA (Steinum et al. 2016)		L2	HT_3	V. anguillarum	Atlantic Salmon (Salmonid)	Norway	unknown	O2a

**NVI4614**	MLSA	MLSA (Steinum et al. 2016)	L2	HT_2	V. anguillarum	Pollock (Gadidae)	Norway	unknown	O2b
**NVI4631**	MLSA	MLSA (Steinum et al. 2016)	L41	HT_28	V. anguillarum	Atlantic Salmon (Salmonid)	Norway	unknown	O2a
**NVI4750**	MLSA	MLSA (Steinum et al. 2016)	L2	HT_2	V. anguillarum	Atlantic cod (Gadidae)	Norway	unknown	O2b
**NVI4792**	MLSA	MLSA (Steinum et al. 2016)	L2	HT_3	V. anguillarum	Atlantic cod (Gadidae)	Norway	unknown	O2a
**NVI4845**	MLSA	MLSA (Steinum et al. 2016)	L41	HT_29	V. anguillarum	Atlantic cod (Gadidae)	Norway	unknown	O2a biotype II
**NVI5022**	MLSA	MLSA (Steinum et al. 2016)	L41	HT_28	V. anguillarum	Atlantic cod (Gadidae)	Norway	unknown	O2a biotype II
**NVI5031**	MLSA	MLSA (Steinum et al. 2016)	L2	HT_3	V. anguillarum	Atlantic cod (Gadidae)	Norway	unknown	O2a biotype II
**NVI5034**	MLSA	MLSA (Steinum et al. 2016)	L2	HT_3	V. anguillarum	Atlantic cod (Gadidae)	Norway	unknown	O2a biotype II
**NVI5039**	MLSA	MLSA (Steinum et al. 2016)	L41	HT_29	V. anguillarum	Atlantic cod (Gadidae)	Norway	unknown	O2a biotype II
**NVI5042**	MLSA	MLSA (Steinum et al. 2016)	L2	HT_2	V. anguillarum	Atlantic cod (Gadidae)	Norway	unknown	O2b
**NVI5043**	MLSA	MLSA (Steinum et al. 2016)	L2	HT_2	V. anguillarum	Atlantic cod (Gadidae)	Norway	unknown	O2b
**NVI5056**	MLSA	MLSA (Steinum et al. 2016)	L41	HT_29	V. anguillarum	Atlantic cod (Gadidae)	Norway	unknown	O2a biotype II
**NVI5064**	MLSA	MLSA (Steinum et al. 2016)	L2	HT_3	V. anguillarum	Pollock (Gadidae)	Norway	unknown	O2a
**NVI5065**	MLSA	MLSA (Steinum et al. 2016)	L2	HT_3	V. anguillarum	Atlantic cod (Gadidae)	Norway	unknown	O2a
**NVI5067**	MLSA	MLSA (Steinum et al. 2016)	L2	HT_2	V. anguillarum	Atlantic cod (Gadidae)	Norway	unknown	O2b
**NVI5070**	MLSA	MLSA (Steinum et al. 2016)	L2	HT_3	V. anguillarum	Rainbow trout (Salmonid)	Norway	unknown	O2a
**NVI5106**	MLSA	MLSA (Steinum et al. 2016)	L2	HT_2	V. anguillarum	Atlantic cod (Gadidae)	Norway	unknown	O2b
**NVI5121**	MLSA	MLSA (Steinum et al. 2016)	L41	HT_29	V. anguillarum	Atlantic cod (Gadidae)	Norway	unknown	O2a biotype II
**NVI5180**	MLSA	MLSA (Steinum et al. 2016)	L1	HT_4	V. anguillarum	Rainbow trout (Salmonid)	Norway	unknown	O1
**NVI5356**	MLSA	MLSA (Steinum et al. 2016)	L2	HT_2	V. anguillarum	Atlantic cod (Gadidae)	Ireland	unknown	O2b
**NVI5543**	MLSA	MLSA (Steinum et al. 2016)	L2	HT_2	V. anguillarum	Atlantic cod (Gadidae)	Norway	unknown	O2b
**NVI5546**	MLSA	MLSA (Steinum et al. 2016)	L2	HT_2	V. anguillarum	Atlantic cod (Gadidae)	Norway	unknown	O2b
**NVI5589**	MLSA	MLSA (Steinum et al. 2016)	L1	HT_4	V. anguillarum	Atlantic Salmon (Salmonid)	Norway	unknown	O1
**NVI6036**	MLSA	MLSA (Steinum et al. 2016)	L41	HT_29	V. anguillarum	Atlantic cod (Gadidae)	Norway	unknown	O2a biotype II
**NVI6037**	MLSA	MLSA (Steinum et al. 2016)	L41	HT_29	V. anguillarum	Atlantic cod (Gadidae)	Norway	unknown	O2a biotype II
**NVI6078**	MLSA	MLSA (Steinum et al. 2016)	L2	HT_2	V. anguillarum	Atlantic cod (Gadidae)	Norway	unknown	O2b
**NVI6099**	MLSA	MLSA (Steinum et al. 2016)	L2	HT_32	V. anguillarum	Atlantic cod (Gadidae)	Norway	unknown	O2b
**NVI6100**	MLSA	MLSA (Steinum et al. 2016)	L2	HT_2	V. anguillarum	Atlantic cod (Gadidae)	Norway	unknown	O2b
**NVI6168**	MLSA	MLSA (Steinum et al. 2016)	L2	HT_2	V. anguillarum	Atlantic cod (Gadidae)	Norway	unknown	O2b
**NVI6243**	MLSA	MLSA (Steinum et al. 2016)	L2	HT_2	V. anguillarum	Atlantic cod (Gadidae)	Norway	unknown	O2b
**NVI6258**	MLSA	MLSA (Steinum et al. 2016)	L1	HT_33	V. anguillarum	Rainbow trout (Salmonid)	Norway	unknown	O1
**NVI6273**	MLSA	MLSA (Steinum et al. 2016)	L2	HT_2	V. anguillarum	Atlantic cod (Gadidae)	Norway	unknown	O2b
**NVI6331**	MLSA	MLSA (Steinum et al. 2016)	L2	HT_2	V. anguillarum	Atlantic cod (Gadidae)	Ireland	unknown	O2b
**NVI6396**	MLSA	MLSA (Steinum et al. 2016)	L2	HT_2	V. anguillarum	Atlantic cod (Gadidae)	Norway	unknown	O2b
**NVI6398**	MLSA	MLSA (Steinum et al. 2016)	L2	HT_2	V. anguillarum	Atlantic cod (Gadidae)	Norway	unknown	O2b
**NVI6401**	MLSA	MLSA (Steinum et al. 2016)	L2	HT_3	V. anguillarum	Atlantic cod (Gadidae)	Norway	unknown	O2a biotype II
**NVI6404**	MLSA	MLSA (Steinum et al. 2016)	L2	HT_3	V. anguillarum	Atlantic cod (Gadidae)	Norway	unknown	O2a
**NVI6409**	MLSA	MLSA (Steinum et al. 2016)	L41	HT_29	V. anguillarum	Atlantic cod (Gadidae)	Norway	unknown	O2a biotype II
**NVI6419**	MLSA	MLSA (Steinum et al. 2016)	L2	HT_2	V. anguillarum	Atlantic cod (Gadidae)	Norway	unknown	O2b
**Se_1_1**	MLSA	MLSA (Steinum et al. 2016)	L2	HT_3	V. anguillarum	Sediment (Sediment)	Denmark	unknown	O2c
**VIB149**	MLSA	MLSA (Steinum et al. 2016)	Other	HT_36	V. anguillarum	Seawater (Seawater)	Denmark	unknown	unknown
**VIB605**	MLSA	MLSA (Steinum et al. 2016)	L1	HT_4	V. anguillarum	Ayu (Plecoglossidae)	Japan	unknown	O1

*** HT assigned on the basis of WGS data**
**** Species on the designation of Steinem et al. not NCBI**
*****Assembly is publicly available but was not used here as we provided our own data for these strains**

Table S2: Quality Control figures for read QC, mapping and assembly of 105 WGS isolates

	Data		Read QC and trimming			
**ID**	**Data Source**	**Assembly level**	**No. reads pre trim**	**No. reads post trim**	**Coverage post trim**	**Mean read length**
**5240-C2**	This Publication	Draft Genome	591499	577924	64.913681	227.567368
**NCIMB6tVib72IOA**	This Publication	Draft Genome	957871	826497	92.335159	226.344681
**Vib1**	This Publication	Draft Genome	344246	283474	31.272411	223.507767
**Vib10**	This Publication	Draft Genome	547697	469916	47.121841	203.163879
**Vib104**	This Publication	Draft Genome	471971	446393	48.916847	222.016647
**Vib109**	This Publication	Draft Genome	312965	278978	29.938038	217.419174
**Vib110**	This Publication	Draft Genome	534057	456215	45.295765	201.155792
**Vib113**	This Publication	Draft Genome	1102286	973462	106.990662	222.674942
**Vib114**	This Publication	Draft Genome	493745	427633	47.269325	223.950821
**Vib115**	This Publication	Draft Genome	605531	487748	54.604137	226.816437
**Vib117**	This Publication	Draft Genome	1019188	898024	98.958244	223.258759
**Vib118**	This Publication	Draft Genome	821619	708737	77.876953	222.622131
**Vib119**	This Publication	Draft Genome	265781	232621	25.160398	219.135681
**Vib122**	This Publication	Draft Genome	732852	618473	65.963539	216.086533
**Vib124**	This Publication	Draft Genome	308634	270562	29.716652	222.524353
**Vib125**	This Publication	Draft Genome	432518	380698	41.377796	220.207047
**Vib126**	This Publication	Draft Genome	832403	727199	77.355667	215.517899
**Vib127**	This Publication	Draft Genome	508507	477125	48.90979	207.686417
**Vib128**	This Publication	Draft Genome	483360	447043	48.670399	220.57692
**Vib13**	This Publication	Draft Genome	416572	347594	37.22805	216.991394
**Vib131**	This Publication	Draft Genome	567971	501853	54.836559	221.379883
**Vib132**	This Publication	Draft Genome	547365	465817	47.918335	208.415909
**Vib134**	This Publication	Draft Genome	533861	451237	45.81184	205.692047
**Vib139**	This Publication	Draft Genome	529708	456109	44.546894	197.876068
**Vib166**	This Publication	Draft Genome	448062	354156	34.48175	197.260071
**Vib175**	This Publication	Draft Genome	704999	585151	60.901119	210.863678
**Vib2**	This Publication	Draft Genome	541528	509640	58.821915	233.840714
**Vib203**	This Publication	Draft Genome	594738	510652	52.563511	208.546936
**Vib222**	This Publication	Draft Genome	526668	447019	45.886444	207.971054
**Vib25**	This Publication	Draft Genome	509011	441503	44.825542	205.700974
**Vib252**	This Publication	Draft Genome	490159	407680	42.256893	210.001602
**Vib253**	This Publication	Draft Genome	796483	671309	66.394569	200.380096
**Vib3**	This Publication	Draft Genome	501898	436436	45.34074	210.48082

	Data		Read QC and trimming			
**ID**	**Data Source**	**Assembly level**	**No. reads pre trim**	**No. reads post trim**	**Coverage post trim**	**Mean read length**
**Vib32**	This Publication	Draft Genome	525389	447137	45.773167	207.402908
**Vib37**	This Publication	Draft Genome	227594	182507	18.617411	206.673233
**Vib39**	This Publication	Draft Genome	413508	341214	37.758434	224.197922
**Vib4**	This Publication	Draft Genome	318154	272399	28.577343	212.54985
**Vib41**	This Publication	Draft Genome	687312	617098	59.448975	195.179718
**Vib44**	This Publication	Draft Genome	263875	235415	25.132265	216.292755
**Vib45**	This Publication	Draft Genome	266376	242828	22.190447	185.144913
**Vib5**	This Publication	Draft Genome	246143	200460	22.155863	223.926468
**Vib53**	This Publication	Draft Genome	521128	470084	46.439693	200.151276
**Vib54**	This Publication	Draft Genome	502343	424403	45.273083	216.125534
**Vib552**	This Publication	Draft Genome	599124	516388	52.346531	205.379105
**Vib553**	This Publication	Draft Genome	630953	542377	55.569424	207.57695
**Vib56**	This Publication	Draft Genome	462155	438193	43.046959	199.031372
**Vib6**	This Publication	Draft Genome	412939	358164	38.065102	215.322571
**Vib60**	This Publication	Draft Genome	351418	305965	32.498322	215.195724
**Vib604**	This Publication	Draft Genome	660864	561006	57.182941	206.511139
**Vib608**	This Publication	Draft Genome	555121	462483	47.937088	210.000504
**Vib61**	This Publication	Draft Genome	408497	344434	37.487774	220.509918
**Vib64**	This Publication	Draft Genome	457891	424527	47.462452	226.511017
**Vib69**	This Publication	Draft Genome	637952	549206	58.48674	215.757858
**Vib7**	This Publication	Draft Genome	466031	403014	42.686707	214.593719
**Vib77**	This Publication	Draft Genome	643499	529999	54.339634	207.72374
**Vib79**	This Publication	Draft Genome	609217	538002	53.107731	199.994629
**Vib8**	This Publication	Draft Genome	507262	479835	47.558334	200.807159
**Vib82**	This Publication	Draft Genome	584909	511370	52.082016	206.346466
**Vib85**	This Publication	Draft Genome	510081	478455	56.234337	238.124985
**Vib86**	This Publication	Draft Genome	576334	493055	49.308857	202.61615
**Vib87**	This Publication	Draft Genome	703696	626903	68.42524	221.136505
**Vib88**	This Publication	Draft Genome	1100053	967195	105.556473	221.11351
**Vib9**	This Publication	Draft Genome	395518	341355	35.608467	211.344757
**Vib93**	This Publication	Draft Genome	238132	201225	22.580015	227.345703
**425**	Publically available assembly	Closed Genome	NA	NA	NA	NA
**775**	Publically available assembly	Closed Genome	NA	NA	NA	NA
**90-11-286**	Publically available assembly	Closed Genome	NA	NA	NA	NA
**CNEVANB11008**	Publically available assembly	Closed Genome	NA	NA	NA	NA
**JLL237**	Publically available assembly	Closed Genome	NA	NA	NA	NA

	Data		Read QC and trimming			
ID	Data Source	Assembly level	No. reads pre trim	No. reads post trim	Coverage post trim	Mean read length
**M3**	Publically available assembly	Closed Genome	NA	NA	NA	NA
**MHK3**	Publically available assembly	Closed Genome	NA	NA	NA	NA
**NB10**	Publically available assembly	Closed Genome	NA	NA	NA	NA
**S3-4-9**	Publically available assembly	Closed Genome	NA	NA	NA	NA
**Vib12**	Publically available assembly	Closed Genome	NA	NA	NA	NA
**VIB43**	Publically available assembly	Closed Genome	NA	NA	NA	NA
**12B09**	Publically available assembly	Draft Genome	NA	NA	NA	NA
**96F**	Publically available assembly	Draft Genome	NA	NA	NA	NA
**ATCC14181**	Publically available assembly	Draft Genome	NA	NA	NA	NA
**FF-93**	Publically available assembly	Draft Genome	NA	NA	NA	NA
**FS-144**	Publically available assembly	Draft Genome	NA	NA	NA	NA
**FS-238**	Publically available assembly	Draft Genome	NA	NA	NA	NA
**HI618**	Publically available assembly	Draft Genome	NA	NA	NA	NA
**M93**	Publically available assembly	Draft Genome	NA	NA	NA	NA
**RV22**	Publically available assembly	Draft Genome	NA	NA	NA	NA
**4299**	Publically available assembly	Scaffold	NA	NA	NA	NA
**51-82-2**	Publically available assembly	Scaffold	NA	NA	NA	NA
**601-90**	Publically available assembly	Scaffold	NA	NA	NA	NA
**6018-1**	Publically available assembly	Scaffold	NA	NA	NA	NA
**87-9-116**	Publically available assembly	Scaffold	NA	NA	NA	NA
**90-11-287**	Publically available assembly	Scaffold	NA	NA	NA	NA
**9014-8**	Publically available assembly	Scaffold	NA	NA	NA	NA
**91-7154**	Publically available assembly	Scaffold	NA	NA	NA	NA
**Ba35**	Publically available assembly	Scaffold	NA	NA	NA	NA
**DSM21597**	Publically available assembly	Scaffold	NA	NA	NA	NA
**HI610**	Publically available assembly	Scaffold	NA	NA	NA	NA
**J360**	Publically available assembly	Scaffold	NA	NA	NA	NA
**LMG12010**	Publically available assembly	Scaffold	NA	NA	NA	NA
**NCTC12159**	Publically available assembly	Scaffold	NA	NA	NA	NA
**PF4**	Publically available assembly	Scaffold	NA	NA	NA	NA
**PF430-3**	Publically available assembly	Scaffold	NA	NA	NA	NA
**PF7**	Publically available assembly	Scaffold	NA	NA	NA	NA
**S22-9**	Publically available assembly	Scaffold	NA	NA	NA	NA
**T265**	Publically available assembly	Scaffold	NA	NA	NA	NA
**VA1**	Publically available assembly	Scaffold	NA	NA	NA	NA
**VIB18**	Publically available assembly	Scaffold	NA	NA	NA	NA

ID	Assembly QC (reference 775)						Mapping QC (reference 775)						
	No. contigs	N50	Assembly length	GC	Reference GC	Ns per 100kbp	% reads mapped	snps	insertion	deletion	Ns	% Ns	Reference length 775
**5240-C2**	167	83901	4090302	44.34	44.51	0	87.11	331	99	118	105883	2.61	4052047
**NCIMB6tVib72IOA**	135	98213	4155178	44.4	44.51	0	76.66	32648	269	284	496357	12.25	4052047
**Vib1**	124	97628	4236491	44.3	44.51	0	86.76	669	110	110	117017	2.89	4052047
**Vib10**	96	240852	3825943	44.56	44.51	0	88.06	31156	253	261	569650	14.06	4052047
**Vib104**	261	50084	4185314	44.4	44.51	0	69.26	32407	255	273	500453	12.35	4052047
**Vib109**	260	43033	4130845	44.44	44.51	0	71.19	31265	218	247	633181	15.63	4052047
**Vib110**	348	25506	4333000	44.43	44.51	0	70.46	31979	220	230	667371	16.47	4052047
**Vib113**	118	106583	4163078	44.42	44.51	0	78.64	30538	241	274	486841	12.01	4052047
**Vib114**	229	73070	4430474	44.62	44.51	0	66.2	31221	226	263	600127	14.81	4052047
**Vib115**	158	101462	4044479	44.49	44.51	0	83.36	34288	240	263	611746	15.1	4052047
**Vib117**	128	212603	4048033	44.51	44.51	0	82.9	32901	233	296	562092	13.87	4052047
**Vib118**	96	215936	3865860	44.56	44.51	0	85.92	33060	250	256	640444	15.81	4052047
**Vib119**	211	50647	3934832	44.64	44.51	0	82.04	31041	204	200	791375	19.53	4052047
**Vib122**	147	131294	4103201	44.4	44.51	0	79.83	26740	222	242	544000	13.43	4052047
**Vib124**	218	58564	4063084	44.45	44.51	0	81.24	25911	201	219	634601	15.66	4052047
**Vib125**	93	252147	4002707	44.59	44.51	0	79.08	33037	257	273	601897	14.85	4052047
**Vib126**	164	71229	4271297	44.42	44.51	0	81.89	316	102	109	159040	3.92	4052047
**Vib127**	93	148998	4201841	44.43	44.51	0	77.22	28936	241	278	525375	12.97	4052047
**Vib128**	91	415033	4047196	44.38	44.51	0	84.48	26643	234	251	527741	13.02	4052047
**Vib13**	213	56522	4117613	44.43	44.51	0	70.09	32076	225	257	592735	14.63	4052047
**Vib131**	174	94934	4492783	44.11	44.51	0	58.2	32646	258	273	475396	11.73	4052047
**Vib132**	295	30883	4124237	44.55	44.51	0	79.98	29681	221	243	569523	14.06	4052047
**Vib134**	395	23207	4173581	44.56	44.51	0	85.73	293	89	91	247230	6.1	4052047
**Vib139**	323	28090	4115570	44.58	44.51	0	79.45	29510	209	244	587098	14.49	4052047
**Vib166**	343	21542	3977898	44.64	44.51	0	78.7	27846	196	237	661973	16.34	4052047
**Vib175**	315	33410	4555784	44.46	44.51	0	75.22	780	100	108	167910	4.14	4052047
**Vib2**	146	99286	4198900	44.34	44.51	0	75.6	32531	261	273	481814	11.89	4052047
**Vib203**	225	33956	3972353	44.69	44.51	0	79.26	30123	222	237	637837	15.74	4052047
**Vib222**	258	29338	3905009	44.85	44.51	0	81.36	29754	208	232	679434	16.77	4052047
**Vib25**	313	30921	4149718	44.41	44.51	0	89.52	319	92	89	232300	5.73	4052047
**Vib252**	269	33081	4040130	44.51	44.51	0	92.87	286	93	94	194155	4.79	4052047
**Vib253**	270	31724	4037812	44.58	44.51	0	92.58	301	95	93	230573	5.69	4052047
**Vib3**	119	106580	4175663	44.34	44.51	0	77.62	30258	233	273	499088	12.32	4052047

ID	Assembly QC (reference 775)						Mapping QC (reference 775)						
	No. contigs	N50	Assembly length	GC	Reference GC	Ns per 100kbp	% reads mapped	snps	insertion	deletion	Ns	% Ns	Reference length 775
**Vib32**	236	40270	4014164	44.56	44.51	0	92.11	263	92	97	238100	5.88	4052047
**Vib37**	149	75712	4016754	44.59	44.51	0	81.98	27106	191	177	990724	24.45	4052047
**Vib39**	118	102931	4090827	44.36	44.51	0	92.83	362	102	107	111054	2.74	4052047
**Vib4**	115	173874	3948396	44.63	44.51	0	80.81	31142	227	231	586276	14.47	4052047
**Vib41**	127	153423	4056929	44.54	44.51	0	76.91	33602	222	276	594804	14.68	4052047
**Vib44**	131	101925	4077185	44.36	44.51	0	89.93	347	96	100	180472	4.45	4052047
**Vib45**	256	60461	4280881	44.54	44.51	0	71.38	29741	192	231	791458	19.53	4052047
**Vib5**	102	135977	3929284	44.58	44.51	0	80.5	31234	217	216	771554	19.04	4052047
**Vib53**	133	136829	4284244	44.2	44.51	0	71.92	29467	245	286	509348	12.57	4052047
**Vib54**	121	264741	3885693	44.59	44.51	0	83.62	31029	243	251	574421	14.18	4052047
**Vib552**	234	39141	3873659	44.66	44.51	0	87.3	30476	224	244	624339	15.41	4052047
**Vib553**	233	43724	4305238	44.39	44.51	0	72.66	29307	221	246	601229	14.84	4052047
**Vib56**	114	99787	4249667	44.31	44.51	0	80.29	320	102	108	71194	1.76	4052047
**Vib6**	124	173462	4181488	44.57	44.51	0	74.25	33589	248	263	585015	14.44	4052047
**Vib60**	102	115571	4118865	44.5	44.51	0	78.08	30041	221	259	526303	12.99	4052047
**Vib604**	308	27897	4116780	44.56	44.51	0	79.11	29738	217	245	573575	14.16	4052047
**Vib608**	321	27536	3955025	44.67	44.51	0	83.2	30181	220	241	642862	15.87	4052047
**Vib61**	120	119467	4126056	44.49	44.51	0	77.91	30207	229	265	507647	12.53	4052047
**Vib64**	136	100623	4066084	44.37	44.51	0	90.98	386	96	109	103005	2.54	4052047
**Vib69**	92	402698	3902957	44.58	44.51	0	85.73	31101	243	259	567403	14	4052047
**Vib7**	136	168216	4105015	44.39	44.51	0	79.58	26696	225	236	544222	13.43	4052047
**Vib77**	215	48569	3906217	44.59	44.51	0	85.9	30774	227	237	620226	15.31	4052047
**Vib79**	122	112342	4099843	44.36	44.51	0	88.63	374	105	113	93034	2.3	4052047
**Vib8**	84	152977	4050283	44.55	44.51	0	79.63	33885	247	262	610206	15.06	4052047
**Vib82**	80	554926	4026164	44.43	44.51	0	76.55	33141	245	300	570832	14.09	4052047
**Vib85**	108	114798	4240372	44.2	44.51	0	86.79	338	105	109	81985	2.02	4052047
**Vib86**	293	31063	4061934	44.48	44.51	0	90.94	352	90	100	188476	4.65	4052047
**Vib87**	133	97506	4045826	44.42	44.51	0	81.6	288	105	102	93396	2.3	4052047
**Vib88**	115	108699	4187861	44.35	44.51	0	90.12	334	104	111	92323	2.28	4052047
**Vib9**	141	115163	4099263	44.36	44.51	0	75.57	33644	230	262	587103	14.49	4052047
**Vib93**	272	35846	4202838	44.45	44.51	0	87.37	229	79	84	448359	11.06	4052047
**425**	3	3164924	4373373	44.44	44.51	0	NA	352	103	112	124013	3.06	4052047
**775**	2	3063912	4052047	44.51	44.51	0	NA	0	0	0	1	0	4052047
**90-11-286**	2	3048854	4342224	44.43	44.51	0	NA	33547	268	271	528328	13.04	4052047
**CNEVANB11008**	2	3132527	4256429	44.58	44.51	0	NA	30192	242	271	503061	12.41	4052047
**JLL237**	2	3122822	4286989	44.44	44.51	0	NA	34401	270	266	502895	12.41	4052047

	Assembly QC (reference 775)						Mapping QC (reference 775)						
**ID**	**No. contigs**	**N50**	**Assembly length**	**GC**	**Reference GC**	**Ns per 100kbp**	**% reads mapped**	**snps**	**insertion**	**deletion**	**Ns**	**% Ns**	**Reference length 775**
**M3**	3	3063587	4117885	44.48	44.51	0	NA	1459	202	92	2256	0.06	4052047
**MHK3**	2	2906233	4015925	44.69	44.51	0	NA	31063	256	318	563367	13.9	4052047
**NB10**	3	3119695	4373835	44.37	44.51	0	NA	326	137	149	64199	1.58	4052047
**S3-4-9**	2	2955425	4182973	44.54	44.51	0	NA	33115	243	263	531608	13.12	4052047
**Vib12**	3	3323092	4897690	44.5	44.51	0	NA	30961	238	290	539395	13.31	4052047
**VIB43**	3	3239943	4407865	44.55	44.51	0	NA	32123	238	273	575323	14.2	4052047
**12B09**	29	1126015	4090643	44.57	44.51	1274	NA	29016	251	263	601907	14.85	4052047
**96F**	141	158521	4044476	44.31	44.51	0	NA	5330	146	163	272719	6.73	4052047
**ATCC14181**	214	57813	4014000	44.48	44.51	0	NA	31859	239	276	661290	16.32	4052047
**FF-93**	214	45726	3833136	44.62	44.51	2	NA	27191	245	261	617018	15.23	4052047
**FS-144**	237	47941	4004106	44.44	44.51	7	NA	28959	251	269	633665	15.64	4052047
**FS-238**	237	50308	3880092	44.56	44.51	6	NA	29254	237	275	604280	14.91	4052047
**HI618**	197	48878	3859630	44.56	44.51	0	NA	31745	233	277	707592	17.46	4052047
**M93**	93	104477	3968196	44.63	44.51	91	NA	31560	239	267	705406	17.41	4052047
**RV22**	235	58580	3993392	44.45	44.51	0	NA	31502	543	379	698185	17.23	4052047
**4299**	2	3468040	4443514	44.62	44.51	14971	NA	31311	230	272	707371	17.46	4052047
**51-82-2**	2	3060835	4171757	44.46	44.51	5840	NA	377	110	109	315192	7.78	4052047
**601-90**	3	3060713	4332198	44.46	44.51	3858	NA	376	107	112	238178	5.88	4052047
**6018-1**	3	3060833	4253109	44.45	44.51	5945	NA	511	110	112	317863	7.84	4052047
**87-9-116**	2	3060464	4167530	44.41	44.51	4526	NA	364	106	110	252207	6.22	4052047
**90-11-287**	3	3060914	4218487	44.47	44.51	3581	NA	366	107	111	213248	5.26	4052047
**9014-8**	3	3060807	4142727	44.4	44.51	3781	NA	335	101	107	328024	8.1	4052047
**91-7154**	3	3061930	4258926	44.47	44.51	5763	NA	388	108	105	317679	7.84	4052047
**Ba35**	3	3060902	4120208	44.45	44.51	3782	NA	314	105	111	196402	4.85	4052047
**DSM21597**	2	3326187	4351326	44.43	44.51	5982	NA	31877	265	276	593801	14.65	4052047
**HI610**	2	3440457	4448222	44.49	44.51	8986	NA	31484	236	268	708239	17.48	4052047
**J360**	4	3195088	4545876	44.47	44.51	0	NA	32133	243	279	575141	14.19	4052047
**LMG12010**	3	3060915	4170967	44.43	44.51	6099	NA	386	108	110	307420	7.59	4052047
**NCTC12159**	6	3192191	4416484	44.44	44.51	0	NA	32290	269	285	506001	12.49	4052047
**PF4**	2	3159229	4180865	44.58	44.51	8208	NA	26283	237	265	619034	15.28	4052047
**PF430-3**	2	3190451	4203852	44.62	44.51	9207	NA	26361	238	269	610939	15.08	4052047
**PF7**	2	3123307	4182004	44.52	44.51	6570	NA	26950	245	246	576445	14.23	4052047
**S22-9**	2	2979966	4002492	44.52	44.51	2755	NA	32737	238	267	623438	15.39	4052047
**T265**	3	3060815	4130209	44.44	44.51	3548	NA	330	106	108	180579	4.46	4052047
**VA1**	3	3060699	4191895	44.44	44.51	4718	NA	431	100	115	238374	5.88	4052047
**VIB18**	3	3060756	4248846	44.37	44.51	5625	NA	349	106	98	331832	8.19	4052047

Table S3: Summary of 10 major lineages identified using WGS and MLSA

Lineage	No. Isolates	MLSA only	WGS only	Both WGS and MLSA	CC-HT**	HT's**
L1	55	19	28	8	CC-HT4	HT_4,HT_15,HT_16,HT_17, HT_18,HT_26,HT_33,HT_37, HT_40
L2	49	40	7	2	CC-HT2	HT_2,HT_3, HT_32,HT_23
L3	9	1	7	1		HT_5
L4	11	4	6	1		HT_1
L5	4	1	3	0		HT_9
L6	3	0	2	1		HT_35
L35	4	3	1	0	CC-HT20	HT_20,HT_21
HT_30 *	4	4	0	0		HT_30
CC-HT28 *	10	10	0	0	CC-HT28	HT_27,HT_28,HT_29
HT_14 *	3	3	0	0		HT_14

* Lineage not represented in PopPUNK

** As assigned in Steinum et al 2016

Table S4: Average pairwise diversity for MLSA loci for 189 isolates.

MLSA loci	***atpA***	***ftsZ***	***gapA***	***gyrB***	***mreB***	***pyrH***	***rpoA***	***topA***
Average pairwise diversity	0. 002316	0.01671	0.006416	0.00778	0.008009	0.005095	0.0012782	0.00817
